# Role of self-assembled molecules in halide perovskite optoelectronics: an atomic-scale perspective

**DOI:** 10.1093/nsr/nwaf150

**Published:** 2025-04-24

**Authors:** Xiaoyu Wang, Xue Wang, Xinjiang Wang, Muchen Li, Hanming Li, Yuhao Fu, Lijun Zhang

**Affiliations:** State Key Laboratory of Superhard Materials, College of Physics, Jilin University, Changchun 130012, China; State Key Laboratory of Integrated Optoelectronics, Key Laboratory of Automobile Materials of MOE, College of Materials Science and Engineering, Jilin University, Changchun 130012, China; State Key Laboratory of Integrated Optoelectronics, Key Laboratory of Automobile Materials of MOE, College of Materials Science and Engineering, Jilin University, Changchun 130012, China; State Key Laboratory of Integrated Optoelectronics, Key Laboratory of Automobile Materials of MOE, College of Materials Science and Engineering, Jilin University, Changchun 130012, China; State Key Laboratory of Integrated Optoelectronics, Key Laboratory of Automobile Materials of MOE, College of Materials Science and Engineering, Jilin University, Changchun 130012, China; State Key Laboratory of Superhard Materials, College of Physics, Jilin University, Changchun 130012, China; State Key Laboratory of Integrated Optoelectronics, Key Laboratory of Automobile Materials of MOE, College of Materials Science and Engineering, Jilin University, Changchun 130012, China

**Keywords:** optoelectronics, self-assembled molecules, atomic-scale mechanism, interface engineering, halide perovskites

## Abstract

Despite significant advancements in the study of metal halide perovskites worldwide, the large-scale industrialization of related optoelectronic devices faces ongoing challenges related to efficiency, long-term stability, and environmental and human toxicity. Self-assembled molecules (SAMs) have recently emerged as crucial strategies for enhancing device performance and stability, particularly by mitigating interface-related challenges. This review provides a comprehensive examination of the multifaceted roles of SAMs in enhancing the performance and stability of perovskite optoelectronic devices. We begin by introducing the evolution of SAMs, their unique physicochemical properties and implemented applications in optoelectronic devices. Subsequently, we delve into the diverse beneficial effects of SAMs in perovskite devices and elucidate the underlying atomic-scale mechanisms responsible for these performance enhancements. Finally, we critically analyze the current challenges associated with the rational design and implementation of SAMs in perovskite devices and conclude by outlining promising future research directions.

## Teaser

This review systematically examines how self-assembled molecules enhance the performance and stability of perovskite optoelectronic devices, focusing on their properties, mechanisms, and rational design strategies.

## INTRODUCTION

Metal halide perovskites, as emerging optoelectronic semiconductor materials, have experienced rapid advancements in various applications, including solar cells, photodetectors, light-emitting diodes and lasers [1,2]. These materials are characterized by their tunable bandgap, high optical absorption, low exciton binding energy, high carrier mobility and cost-effectiveness. The unique electronic structure of metal halide perovskites plays a crucial role in their superior performance, featuring strong band-edge dispersion, cross-bandgap hybridization, band-edge antibonding states and Rashba spin splitting [3]. Significant progress in perovskite optoelectronic devices has been achieved through a comprehensive understanding of the structure–property relationship of these materials. Notably, the power conversion efficiency (PCE) of perovskite solar cells (PSCs) has surpassed 26% [4], while the external quantum efficiency (EQE) of perovskite light-emitting diodes (PeLEDs) has exceeded 30% [5], as of 2024. Enhancing cost-effectiveness by improving both efficiency and long-term stability, while simultaneously reducing environmental and human toxicity, is crucial for the large-scale industrialization of perovskite materials. This pathway presents both challenges and research opportunities throughout the entire process, from material design to device synthesis.

**Figure 1.**
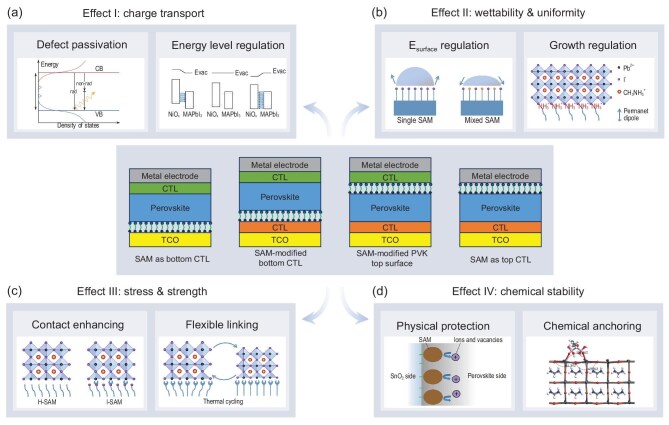
Four modes of SAM application in perovskite optoelectronic devices (middle panel), along with their associated effects and corresponding atomic-scale mechanisms. (a) Optimization of interfacial charge transport through defect passivation and energy level alignment. Adapted with permission from [17] and [16]. Copyright 2020, Royal Society of Chemistry. Copyright 2017, Wiley-VCH. (b) Regulation of interfacial wettability and uniformity via surface energy modulation and control of crystal growth. Adapted with permission from [18] and [19]. Copyright 2019, Wiley-VCH. Copyright 2015, American Chemical Society. (c) Enhancement of interfacial mechanical strength and alleviation of interfacial stress through improved contact and flexible connections. Adapted with permission from [21] and [20]. Copyright 2021, American Association for the Advancement of Science. Copyright 2023, Springer Nature. (d) Improvement of interfacial chemical stability through physical isolation and chemical anchoring. Adapted with permission from [22] and [23]. Copyright 2020, Springer Nature. Copyright 2024, Wiley-VCH. CB: conduction band; VB: valence band; CTL: charge transport layer; TCO: transparent conductive oxide; PVK: perovskite.

PSC and PeLED devices share similar architectures, comprising essential components such as the active layer, charge transport layers and electrodes. Over the past decade, the optimization of PSCs has led to the development of well-established chemical compositions for the active layer. This progression has evolved from A-site cations, such as methylammonium (MA) and formamidinium (FA) [6], to multi-cation and mixed-halide alloys [7], resulting in several widely adopted formulations. In recent years, surface and interface engineering have emerged as key factors influencing device performance. Accordingly, many studies have been conducted focusing on the optimization of transport layers. A range of materials with excellent charge transport properties have been developed for use as electron transport layers, including TiO_2_, ZnO, SnO_2_, C_60_ and PC_61_BM ([6,6]-phenyl-C61-butyric acid methyl ester), as well as hole transport layers such as Spiro-OMeTAD (2,2’,7,7’-tetrakis(N, N’-di-*p*-methoxyphenylamine)-9,9’-spirobifluorene), NiOx, PEDOT: PSS (poly(3,4-ethylenedioxythiophene: polystyrenesulfonate)) and PTAA (poly[bis(4-phenyl) (2,5,6-trimethylphenyl)-amine]). However, these materials face persistent challenges, including high processing temperatures, low stability, poor interface contact quality (interface defects), significant parasitic absorption and high production costs [8,9]. In this context, self-assembled molecules (SAMs) have been successfully applied in perovskite optoelectronic devices. SAMs can serve as transport layers on both sides of the perovskite [10,11] or be used to modify the surface of the bottom transport layer [12,13] and the top surface [14,15] of the perovskite (Fig. 1).

The beneficial effects of SAMs on optoelectronic devices, as observed through experimental methods, are primarily attributed to the atomic-scale interactions between SAMs and the various functional layers within the device (Fig. 1). First, SAMs can introduce additional dipoles to regulate interfacial energy level alignment [16] and passivate interfacial defects [17], thereby optimizing the carrier transport process at the interface. Second, SAM-modified interfaces can influence surface energy [18] and perovskite growth [19], thereby controlling the wettability of perovskite precursor solutions and enhancing the uniformity of perovskite films. Third, the intrinsic flexibility of SAMs, along with the additional interfacial interactions they introduce, can alleviate interfacial stress [20] and strengthen the interfacial bond [21]. Finally, the barrier [22] and anchoring effects [23] of SAMs at the interface can improve the chemical stability of optoelectronic devices by inhibiting undesirable chemical reactions or phase segregation. In recent years, the mechanisms outlined above have been widely applied to single-junction and tandem PSCs, as well as PeLEDs, becoming key factors in enhancing both device efficiency and long-term stability. In the supporting information (Table S1), we summarize major works published between 2018 and 2024 that focus on the design and application of SAM layers. This includes a compilation of the achieved device efficiencies and long-term stability under specific conditions. Through the rational use and optimization of SAMs, the PCE of single-junction PSCs has exceeded 26% [12], the EQE of PeLEDs has approached 20% [24], and the efficiency of perovskite-silicon tandem solar cells has surpassed 32% [25].

In this review, we summarize the diverse beneficial effects of SAMs in perovskite optoelectronic devices, along with the underlying atomic-scale mechanisms. We begin by outlining the evolution of SAMs, molecular structural characteristics, physicochemical properties and implemented applications of SAMs in optoelectronic devices. Next, we review how SAMs regulate and optimize various interfacial properties in perovskite optoelectronic devices, including charge transport, wettability, uniformity, stress, mechanical strength and chemical stability. We then provide a mechanistic explanation for the superior interfacial modification capabilities of SAMs by summarizing the atomic-scale physical and chemical processes underlying each effect. Finally, we discuss the challenges faced by SAMs in characterization, usage and design, and provide corresponding potential solutions and valuable research directions for each.

## OVERVIEW OF SAMS

### Evolution of SAMs

The term most commonly associated with the abbreviation SAMs is ‘self-assembled monolayers’. However, in this context, we expand the concept to ‘self-assembled molecules’ to highlight that, in some contemporary applications, the formation of a strict monolayer is not always observed [26], and amorphization may even occur [27]. The research on SAMs has a long history, with the field gaining significant attention even before its application in the physical modification of optoelectronic devices. As early as the 1940s, researchers successfully synthesized SAM monolayers [28]. Although the practical applications of SAMs were not fully realized at that time, their theoretical significance in surface modification and the integration of 2D-3D materials was already apparent [29]. In subsequent studies, SAMs were rapidly adopted for the electrical and optical modification of material surfaces, leading to numerous practical applications, including corrosion protection, lubrication, sensors and electronic devices [30]. In 2014, SAMs were employed to enhance charge transport in PSCs [31], marking the beginning of their widespread use in perovskite optoelectronic devices and establishing them as a crucial method of interfacial modification. Over the past five years, SAMs have played a pivotal role in research focused on efficiency breakthroughs in both PSCs and PeLEDs.

### Molecular structure of SAMs

The fundamental structural characteristics of SAMs can be categorized into three components: the terminal group, the linker group and the anchoring group. In an ideal scenario, the anchoring group facilitates the chemical adsorption of SAMs onto the substrate, while the linker and terminal groups promote the self-assembly process of the monolayer molecules, ultimately leading to the spontaneous formation of a dense and stable self-assembled monolayer on the substrate.

**Figure 2.**
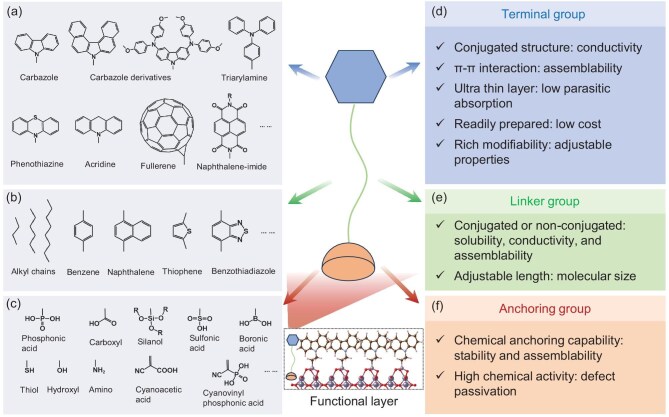
Representative examples of terminal groups, linker groups and anchoring groups in SAMs as reported in the literature (a–c), along with the intrinsic advantages of each type of functional group (d–f).

When used as a transport or modification layer at the buried interface, the terminal groups of SAMs are typically in direct contact with the perovskite layer. Conversely, when applied as a modification or transport layer on the surface of perovskite, the terminal groups interact with the transport layer or electrode material. As the key groups influencing the properties of the modified surface, the terminal groups play a critical role in controlling wettability, regulating crystal growth, improving charge transport, modulating energy band alignment, passivating surface defects and enhancing interfacial stability and long-term device reliability. SAMs used in optoelectronic devices typically require good conductivity, which is provided by conjugated structures (Fig. 2a) such as carbazole and its derivatives [32–35], triphenylamine and its derivatives [36], phenothiazine [37], acridine [38], naphthalene-imide [39,40] and fullerenes [41], among others. In addition to enhancing conductivity, conjugated structures can promote the self-assembly process through intermolecular π-π interactions. However, excessive aggregation must be avoided, as it can lead to inhomogeneity [42]. As the component with the highest mass fraction in SAMs, the terminal functional groups generally demonstrate relatively low molecular weights and structural complexity (typically exhibiting lower values compared to benchmark materials such as Spiro-OMeTAD). This inherent simplicity facilitates the formation of thin, structurally simple layered configurations. Consequently, SAMs present reduced synthetic complexity and comparatively lower manufacturing costs. Building on the structures shown in Fig. 2a, terminal groups can be further modified by introducing a variety of functional groups at different positions on the phenyl group, thereby increasing the degree of control over the properties of the terminal groups. By rationally designing these terminal groups, the efficiency, stability and other properties of perovskite optoelectronic devices can be optimized.

The linker groups in SAMs serve as the molecular backbone, connecting the anchoring group to the terminal group. Common linker groups include alkyl chains and aromatic conjugated groups, which modulate intramolecular interactions between the terminal group and the anchoring group (substrate), intermolecular interactions (e.g. van der Waals forces) and interactions with the solvent (e.g. solubility). Representative linker groups (Fig. 2b) reported in the literature include alkyl chains, benzene [36], naphthalene [43], thiophene [44], benzothiadiazole [45] and others. For linker groups with conjugated structures, photogenerated electrons can migrate along the longitudinal axis of the molecule through the conjugated π-electron system. In contrast, linker groups with non-conjugated structures may inhibit or decouple electron migration between the upper and lower portions of the molecule, thereby affecting the efficiency and pathway of charge transport. For example, Levine *et al.* found that increasing the length of the alkyl chain in SAMs enlarges the tunneling barrier, thereby leading to lower hole transport efficiency [46]. The anchoring and terminal groups together determine the layer thickness and molecular geometry when the SAM forms a layered structure.

Anchoring groups play a crucial role in the molecular design of SAMs and are involved in the bonding interactions between each molecule and the substrate through chemical reactions such as hydrolysis and condensation, thereby determining the bonding strength between the substrate and the SAM. Various anchoring groups (Fig. 2c), including phosphonic acid [33], carboxyl [36], silanol [21], sulfonic acid [47], boronic acid [48], thiol [49], hydroxyl [50] and amino [51], have been developed to anchor onto metal oxides and other substrates such as indium tin oxide (ITO), fluorine-doped tin oxide (FTO), NiO_x_, TiO_2_ and SnO_2_ through different types of interactions. The anchoring process influences the tilt angle of the molecule with respect to the surface normal vector, the work function of the substrate, interfacial dipoles, contact resistance and interfacial charge transfer, thus significantly impacting the electronic function and performance stability of perovskite optoelectronic devices [52,53]. When in contact with perovskite, the chemical activity of the anchoring groups imparts defect passivation capabilities, and the ionization and protonation of anchoring groups can also affect their passivation efficiency for different types of defects [54]. Further modification of the anchoring groups can also enhance the potential for property tuning, such as by adding cyano groups (e.g. cyanoacetic acid [45] and cyanovinyl phosphonic acid [55]).

### Physicochemical properties of SAMs

Due to the inherently ‘modular’ structure of SAMs, they offer a vast range of controllable physical-chemical properties. The combined functionality of the various components (Fig. 2d–f) of the SAM contributes to its intrinsic advantages. SAMs possess three distinct advantages compared to conventional metal oxide charge transport materials (such as TiO_2_, SnO_2_, ZnO and NiO_x_) and common interfacial modification molecules. Firstly, by introducing uniform molecular dipoles, SAMs effectively tune the work function of the substrate. This capability is critical for optimizing charge transport and energy level alignment in electronic devices. Secondly, SAMs can be prepared via a thermodynamically favorable self-anchoring process from vapor or liquid phases under mild reaction conditions. This allows for scalable and flexible manufacturing while minimizing energy consumption and production costs. Thirdly, the monomolecular thickness of SAM layers leads to minimal material consumption and reduced parasitic absorption, which enhances device efficiency and reduces costs.

SAMs form films with high surface uniformity and structural stability, excellent corrosion and wear resistance, and long-term durability, meeting the requirements for high-performance devices that demand surface accuracy and robustness. Additionally, mechanical stability is another significant advantage of SAMs. Mechanical stress in solution-processed thin-film electronics can lead to severe degradation of device performance. However, the strong covalent bond between the anchoring group and the substrate, coupled with the softer interactions between the linker and terminal groups (e.g. van der Waals forces or π-π interactions), endows SAMs with good tolerance and even self-healing capabilities in response to intermolecular displacement or deformation caused by mechanical stress [56].

### Implemented application of SAMs in optoelectronic devices

Due to the structural flexibility of SAMs, certain SAMs can serve as active materials in optoelectronic devices. SAMs with a donor–π–bridge–acceptor structure, for example, can function as sensitizers in dye-sensitized solar cells (DSCs) [57] and as photoactive materials in organic solar cells [58]. In terms of interfacial modification, SAMs offer a diverse range of functionalities. In the 2000s, SAMs were already being used for the surface modification of ITO electrodes in organic light-emitting diodes and organic solar cells, with the aim of enhancing hydrophobicity [59] and improving charge transport [60,61]. Additionally, SAMs have been employed for the surface modification of oxides in DSCs, improving their binding strength with sensitizers [62,63]. The development of PSCs has been significantly influenced by advancements in organic solar cells and DSCs. Regarding SAM applications, in addition to similar interface modification strategies, various functions specifically tailored for perovskite systems have emerged. In the next section, we provide a detailed overview of the observable effects induced by SAMs in perovskite optoelectronic devices.

## BENEFICIAL EFFECTS OF SAMS IN PEROVSKITE DEVICES

### Improving interfacial charge carrier transport

**Figure 3.**
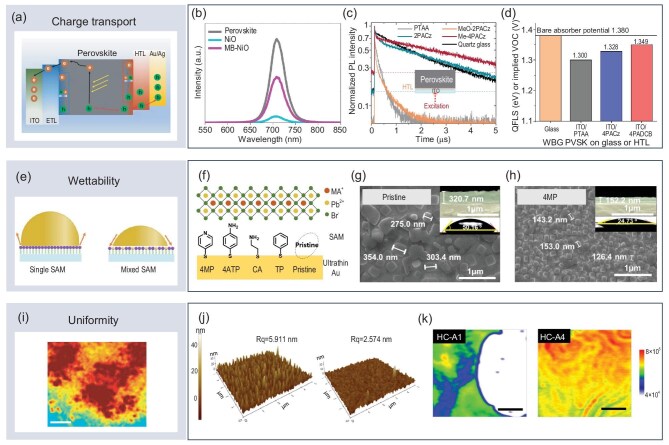
Experimental techniques used to observe the enhancement of charge transport, wettability and uniformity in perovskite optoelectronic devices by SAMs. (a) Schematic diagram of interfacial charge transport. Adapted with permission from [64]. Copyright 2022, Wiley-VCH. (b) Photoluminescence spectra. Adapted with permission from [65]. Copyright 2022, Springer Nature. (c) Time-resolved photoluminescence. Adapted with permission from [67]. Copyright 2020, American Association for the Advancement of Science. (d) Quasi-Fermi level splitting. Adapted with permission from [35]. Copyright 2023, Springer Nature. (e) Schematic diagram of wettability. Adapted with permission from [18]. Copyright 2019, Wiley-VCH. (f–h) Effect of SAM anchoring on the contact angle of the ultrathin Au layer. Adapted with permission from [49]. Copyright 2021, American Chemical Society. (i) Schematic diagram of uniformity. Adapted with permission from [74]. Copyright 2018, Springer Nature. (j) Atomic force microscopy. Adapted with permission from [75]. Copyright 2018, American Chemical Society. (k) Photoluminescence mapping. Adapted from [43], licensed under CC BY 4.0.

The most significant modification that SAMs induce in perovskite optoelectronic devices is the modulation of interfacial charge carrier transport properties (Fig. 3a) [64]. One of the most successful applications is their use as a modification for hole transport layers or as hole transport layers themselves. Steady-state photoluminescence (PL) and quasi-Fermi level splitting (QFLS) are common techniques used to study charge transfer kinetics at interfaces.

In PSCs, primary functions of SAMs include suppressing non-radiative recombination and enhancing interfacial charge transfer, thereby improving overall device performance. Modifying the NiO_x_ hole transport layer [65] in PSCs with SAMs or directly applying SAMs as the hole transport layer [35] can significantly enhance PL (Fig. 3b) and QFLS (Fig. 3d), demonstrating SAMs’ remarkable ability to reduce non-radiative recombination of carriers at the interface. Notably, time-resolved PL (TRPL) is a more intuitive method for characterizing the rate of PL decay. However, due to the multifunctionality of SAMs in enhancing interface charge extraction and suppressing non-radiative recombination, distinguishing the primary factors influencing PL decay is essential. Examining changes in the slope of TRPL provides an effective means of differentiation [66]. Rapid changes in TRPL on short time scales indicate enhanced interface transport, while slower changes on longer time scales reflect the suppression of non-radiative recombination. Recent studies [67] show that SAMs can accelerate interface charge transport while simultaneously suppressing non-radiative recombination processes (Fig. 3c).

Improving interface charge transport with SAMs can significantly enhance the efficiency of PSCs. Many recent breakthroughs in efficiency records for single‐junction and tandem PSCs have been achieved through SAM design. In PeLEDs, the role of SAMs is primarily to enhance radiative efficiency and regulate exciton recombination dynamics. When SAMs are used to modify the hole transport layer [68] of PeLEDs or directly as the hole transport layer [69], they can passivate film defects to increase PL and QFLS intensity, as well as the current at the same excitation voltage and the radiative lifetime as characterized by TRPL. These modifications have also contributed to multiple breakthroughs in achieving record EQE for blue PeLEDs. It is worth noting that the enhancement of interfacial carrier transport by SAMs is not limited to the hole transport interface. For example, modifying SnO_2_ [70] and TiO_2_ [71] electron transport layers with fullerene-derived SAMs has also been proven to be an effective approach for enhancing electron transport and extraction.

### Enhancing wettability and uniformity

The wettability of the substrate primarily influences the perovskite film formation process and is a critical factor in regulating both physical contact at the interface and the morphology of perovskite grains. As an effective surface modification technique, SAMs can efficiently alter the wettability of the substrate. The most commonly used experimental method to measure wettability is the contact angle measurement (Fig. 3e). By using different types of SAMs, the hydrophobicity and hydrophilicity of the substrate surface can be modified, leading to both increases [72] and decreases in the contact angle [43,49]. Kim *et al.* demonstrated continuous modulation of the contact angle by employing different SAMs (Fig. 3f) as the modification layer for ultrathin Au. As the contact angle decreased, the perovskite grain size gradually reduced (Fig. 3g and h) [49]. The reduced grain size enhances the EQE of PeLEDs by confining excitons within grains to increase the radiative recombination rate, reducing surface pinholes to improve the uniformity and coverage of the nanograin layer, and minimizing current leakage while limiting exciton diffusion to suppress exciton dissociation [49,73].

Interface uniformity (Fig. 3i) is a crucial factor in the preparation of large-scale, high-efficiency perovskite devices [74]. SAMs can significantly improve interface uniformity. This enhancement can be categorized into two aspects: one is the improvement in the uniformity of substrate morphology after modification [75], and the other is the macroscopic distribution uniformity of perovskite crystalline films [43]. The improvement in uniformity resulting from the addition of SAMs can be observed through atomic force microscopy (AFM) and PL mapping. AFM clearly shows the differences in surface roughness of the SnO_2_ transport layer before and after SAM modification (Fig. 3j). A reduction in roughness indicates improved substrate uniformity during perovskite deposition [75]. PL mapping provides a clear characterization of the distribution of the deposited perovskite films, including micron-scale voids caused by material deficiencies. These voids can be effectively filled by adjusting the type of SAM used to modify the substrate (Fig. 3k), thereby optimizing the film's uniformity [43].

### Mitigating interfacial stress and enhancing mechanical strength

**Figure 4.**
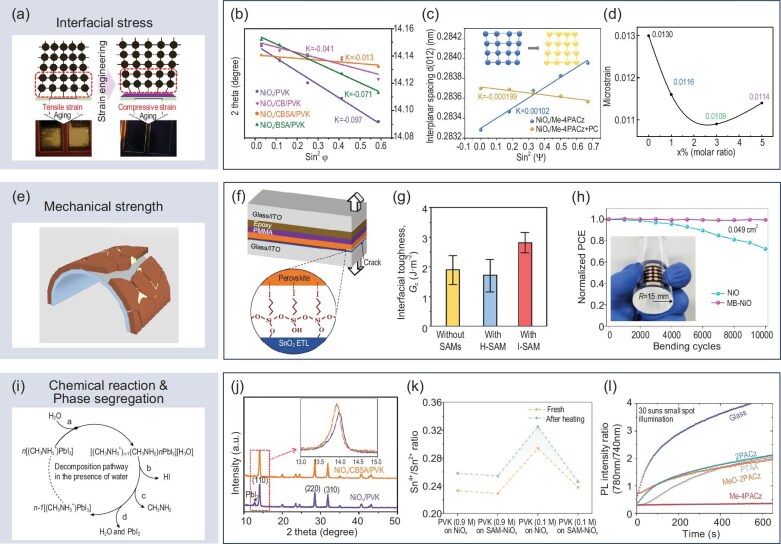
Experimental techniques used to observe the effects of SAMs on interfacial stress release, enhancement of mechanical strength, and suppression of chemical reactions and phase segregation in perovskite optoelectronic devices. (a) Schematic diagram of interfacial stress. Adapted with permission from [76]. Copyright 2022, American Chemical Society. (b, c) Grazing incidence X-ray diffraction. Adapted with permission from [77]. Copyright 2022, Wiley-VCH. Adapted from [78], licensed under CC BY 4.0. (d) Williamson-Hall method. Adapted with permission from [79]. Copyright 2023, Elsevier. (e) Schematic diagram of mechanical strength. Adapted with permission from [80]. Copyright 2021, Elsevier. (f, g) Double-cantilever beam method. Adapted with permission from [21]. Copyright 2021, American Association for the Advancement of Science. (h) Fixed-radius bending method. Adapted with permission from [65]. Copyright 2022, Springer Nature. (i) Schematic diagram of chemical reactions. Adapted from [82], licensed under CC BY 4.0. (j) X-ray diffraction. Adapted with permission from [77]. Copyright 2022, Wiley-VCH. (k) X-ray photoelectron spectroscopy. Adapted with permission from [85]. Copyright 2023, Wiley-VCH. (l) Photoluminescence peak shifts. Adapted with permission from [67]. Copyright 2020, American Association for the Advancement of Science.

During the layer-by-layer spin-coating crystallization process of perovskite optoelectronic devices, interface stress (Fig. 4a) [76] inevitably develops after crystallization due to the mismatch of lattice parameters between different crystalline materials. SAMs, with their flexible molecular structure and potential non-chemical interactions at the terminal groups, can act as a buffer layer, alleviating interface stress. The ratio of the diffraction angle to the sine of the tilt angle in grazing incidence X-ray diffraction (GIXRD) measurements can be used to estimate the residual stress in the material. The addition of SAMs significantly reduces this ratio, effectively regulating interface stress [77,78]. Zhang *et al.* found that modifying the NiO_x_ surface with different SAMs (Fig. 4b) could alleviate the perovskite-NiO_x_ interface stress to varying degrees [77]. Cao *et al.* demonstrated that a co-SAM blending approach (Fig. 4c) allows for more precise control over both the direction and magnitude of the stress [78]. Furthermore, the Williamson-Hall method can estimate residual strain through the diffraction angle and the full width at half maximum of the X-ray diffraction (XRD) peak (Fig. 4d), and the proper incorporation of SAMs can also reduce residual strain [79].

The presence of residual stress can weaken the interface, thereby reducing the overall mechanical stability of optoelectronic devices. Interface mechanical strength (Fig. 4e) [80] plays a crucial role in determining the structural stability of perovskite optoelectronic devices during processes such as transportation, installation and exposure to thermal stress. It is a key factor influencing the device's lifetime, in addition to the intrinsic stability of the functional layer materials. Experimental methods, such as the double-cantilever beam (DCB) method [21,81] or the fixed-radius bending method [65], can be used to measure the mechanical strength of the device. The incorporation of SAMs significantly enhances the stability of devices in both tests. Dai *et al.* found that SAMs with I-terminal groups [21] can increase the interfacial adhesion toughness between the transport layer and the perovskite by 50% (Fig. 4f and g). Li *et al.* demonstrated that modifying the NiO_x_ layer with SAMs [65] enabled the flexible PSC device to maintain a constant PCE after 10 000 bending cycles (Fig. 4h).

### Preventing chemical degradation and phase segregation

One of the key factors influencing the chemical stability of perovskite optoelectronic devices is the detrimental chemical reactions (Fig. 4i) [82] that occur between the active layer and the charge transport layer/electrode. The protonated A-site ions of perovskite and halide ions are chemically reactive, allowing them to interact with metal oxide layers [83]. The introduction of SAMs can help inhibit these reactions. XRD can effectively characterize this inhibitory effect [77,84]. For example, changes in the PbI_2_ peak before and after SAM modification (Fig. 4j) can be used to assess the suppressive effect of SAMs on the detrimental reaction between the perovskite and NiO_x_ [77]. In the case of Sn-based perovskites, Sn is prone to oxidation by under-coordinated metal ions in metal oxides, a process that SAMs can inhibit. X-ray photoelectron spectroscopy (XPS) can be employed to quantify the amount of under-coordinated metal ion reactants (Fig. 4k), thus allowing for the observation of SAMs’ inhibitory effect on this oxidation process [85].

Phase segregation within the perovskite active layer is another significant issue that impacts the long-term stability of perovskite optoelectronic devices. The formation of new phases due to phase segregation can cause shifts in the peak positions of the PL spectrum. However, SAMs can effectively suppress these shifts in peak positions [67,86,87]. Al-Ashouri *et al.* demonstrated a clear suppression of perovskite phase segregation by using SAMs, as evidenced by comparing the PL intensities at 780 nm (corresponding to the I-rich phase formed by phase segregation) and 740 nm (corresponding to the perovskite without phase segregation). The ratio between these intensities remained constant (Fig. 4l), indicating that SAMs effectively inhibit phase segregation [67].

## ATOMIC-SCALE MECHANISMS UNDERLYING BENEFICIAL EFFECTS OF SAMS

### Defect states passivation

The influence of defects on interface charge transfer in perovskite optoelectronic devices arises from disturbances to the electronic structure. The introduction of additional atoms or partial atomic vacancies can lead to localized charge imbalances, changes in the coordination environment and anomalous local bonding, all of which contribute to the formation of isolated defect levels. When these defect levels fall within the bandgap, they act as recombination centers (trapping levels) for carriers, thereby significantly impacting the interface charge transport process [17]. Excessive Pb or under-coordinated Pb^2+^, which forms Pb-Pb dimers, typically results in deeper defect energy levels, severely affecting the interface charge transport [88].

**Figure 5.**
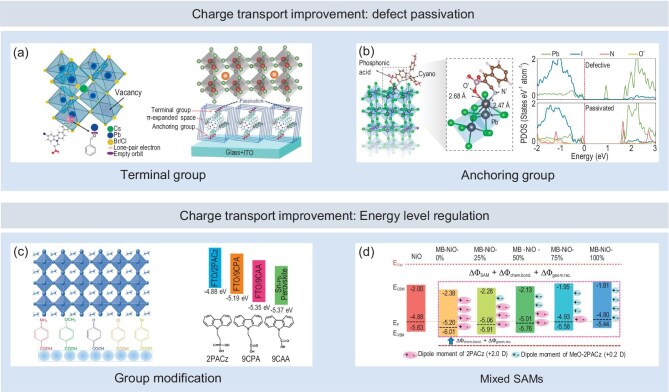
The atomic-scale mechanism by which SAMs optimize charge transport through defect passivation and energy level alignment modulation. (a, b) The defect passivation effects of the terminal groups (a) and anchoring groups (b) of SAMs. Adapted with permission from [69], [89] and [55]. Copyright 2023, Elsevier. Copyright 2023, Wiley-VCH. Copyright 2023, American Association for the Advancement of Science. (c) Modulation of energy level alignment through the design of SAM terminal groups and anchoring groups. Adapted with permission from [16] and [92]. Copyright 2017, Wiley-VCH. Copyright 2024, Wiley-VCH. (d) Continuous modulation of energy level alignment through the mixed use of SAMs with different dipoles. Adapted with permission from [65]. Copyright 2022, Springer Nature.

Filling lattice vacancies and completing coordination with defect atoms to neutralize local charges is a unified approach to eliminating trap states. The introduction of passivators to facilitate additional chemical bonding processes aligns with the principles of Lewis acid-base neutralization. For example, lead interstitial defects in perovskite, acting as a Lewis acid, can be passivated by terminal groups in certain SAMs, which exhibit Lewis basic properties [68,89]. Chen *et al.* designed a (2-(3-bromo-6-(4-formylphenyl)-9H-carbazol-9-yl)ethyl)phosphonic acid SAM [69], which, when anchored on ITO, demonstrated that the carbonyl group in the terminal group exhibited Lewis basicity, effectively passivating under-coordinated Pb^2+^ (Fig. 5a, left panel). Similarly, Hung *et al.* synthesized a symmetric porphyrin SAM with carboxyl groups on both sides [89], which, when anchored on ITO, could coordinate with Pb^2+^ defects in the perovskite (Fig. 5a, right panel).

Interestingly, when SAMs partially flip or form anisotropic layers, their anchoring groups can also exhibit properties of Lewis bases, thereby passivating lead-related defects [55,65]. Li *et al.* were the first to observe the potential partial flipping behavior of SAMs using sum-frequency generation (SFG) spectroscopy, providing evidence for the interaction between the anchoring group (phosphonic acid group) and Pb defects in the perovskite. Additionally, they employed first-principles calculations to investigate the coordination effect of the phosphonic acid group on Pb defects, which effectively eliminates deep defect energy levels [65]. This approach was later applied by Zhang *et al.* in the development of an additional SAM layer with random molecular orientation [55]. Based on an unwashed SAM layer, they created more opportunities for anchoring group-defect interactions. By incorporating a cyano group in addition to the phosphonic acid anchoring group, they further enhanced the defect passivation capabilities of the anchoring groups (Fig. 5b).

Apart from lead-related defects, other defects, such as under-coordinated halide defects and cation vacancies, introduce relatively shallow defect states. However, they still influence carrier transport. The introduction of different SAM terminal groups can also effectively passivate the corresponding shallow defect states. For example, Hou *et al.* utilized the terminal amine group of dopamine molecules to passivate under-coordinated iodine defects through hydrogen bonding interactions [75]. Li *et al.* achieved the filling of cation vacancy defects by introducing CA-Br (6-carboxy-N, N, N-triethylhexan-1-aminium bromide) molecules [18].

### Modulating energy level alignment

In addition to the abundant defects at the interface, another major factor contributing to charge transport issues is the alignment of energy levels. Imperfect energy level alignment can hinder charge transport, leading to charge accumulation at the interface and promoting non-radiative recombination processes. In PSCs, energy level alignment directly influences the QFLS, which, in turn, affects variations in the open-circuit voltage (V_OC_).

The ability of SAMs to modulate energy levels arises from the intrinsic electric dipole they generate when anchored to the substrate. This alters the material's work function, resulting in changes to energy level alignment. As reported by Zhu *et al.*, first-principles calculations of molecular dipoles reveal a clear linear relationship with the experimentally measured work function [90]. Due to their flexible structural composition and excellent self-assembly properties, SAMs enable precise control over energy level alignment. By modifying the functional groups of SAMs to adjust the dipole moment, or by blending different types of SAMs, it is possible to finely tune energy level alignment [16,65,91]. This allows for customized adjustments of energy level alignments at various interfaces in different devices.

Wang *et al.* systematically investigated the effect of replacing terminal groups on the energy level alignment at the NiO_x_-perovskite using a benzoic acid SAM (Fig. 5c, left panel). The variation in anchoring groups induces a continuous change in the SAM's dipole. When amino, methoxy, or hydrogen groups are used as terminal groups, the dipole points from NiO_x_ towards the perovskite. In contrast, when Br or Cl terminal groups are employed, the dipole points from the perovskite towards NiO_x_. Ultimately, they determined that the Br-terminal group achieved the optimal energy level alignment [16]. Similarly, Zhang *et al.* adjusted the energy level alignment between FTO and perovskite by modifying the anchoring and linker groups of a carbazole-based SAM [92], specifically by replacing the phosphonic acid group with a carboxylic acid group and shortening the linker group length (Fig. 5c, right panel).

Due to the significant electronegativity differences among various SAM functional groups, achieving a linear and continuous adjustment of the dipole through the design of functional groups within the SAM alone is not feasible. In this context, continuous modulation of interface energy level alignment can be achieved by using SAMs with differing dipoles simultaneously. Li *et al.* modified the NiO_x_ layer with two SAMs having different dipoles: 2PACz ([2-(9H-carbazol-9-yl)ethyl]phosphonic acid) and MeO-2PACz ([2-(3,6-dimethoxy-9H-carbazol-9-yl)ethyl]phosphonic acid). By adjusting the ratio of these two SAMs, they achieved continuous modulation of the energy level alignment between NiO_x_ and perovskite (Fig. 5d). Ultimately, they determined that a 3 : 1 ratio of 2PACz to MeO-2PACz resulted in the optimal energy level alignment [65].

### Surface energy engineering and crystal growth controlling

The wettability of the perovskite precursor solution on the substrate affects both the interfacial contact quality and the morphology of perovskite grains [49], which, in turn, influence charge transport at the interface and within the perovskite. Nucleation and growth of perovskites on substrates with poor wettability may introduce microscopic voids, which are detrimental to interfacial charge transport [43]. However, some researchers argue that larger perovskite grain sizes can reduce grain boundaries, thereby enhancing the electrical transport properties within the perovskite. This can be achieved through low wettability [72,93].

**Figure 6.**
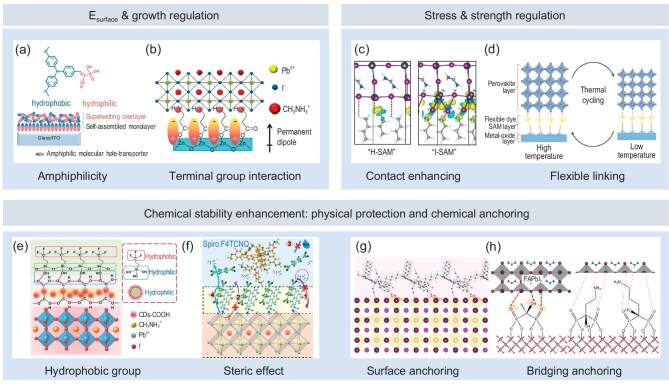
Atomic-scale mechanisms by which SAMs regulate wettability, uniformity, stress and mechanical strength, and enhance chemical stability at the interface. (a) Regulation of surface energy (E_surface_) through amphiphilic properties. Adapted with permission from [55]. Copyright 2023, American Association for the Advancement of Science. (b) Crystal growth regulation. Adapted with permission from [19]. Copyright 2015, American Chemical Society. (c) Enhancement of contact by adding additional interfacial chemical bonds. Adapted with permission from [21]. Copyright 2021, American Association for the Advancement of Science. (d) Stress relief through molecular flexibility. Adapted with permission from [20]. Copyright 2023, Springer Nature. (e) Prevention of moisture-induced corrosion using hydrophobic groups. Adapted with permission from [14]. Copyright 2020, Royal Society of Chemistry. (f) Blocking ion and molecular migration through the steric effects of a densely assembled layer. Adapted with permission from [104]. Copyright 2024, Royal Society of Chemistry. (g, h) Inhibition of ion migration through surface (g) and bridging (h) chemical anchoring. Adapted with permission from [105] and [23]. Copyright 2022, Wiley‐VCH. Copyright 2024, Wiley‐VCH.

Wettability is directly related to surface energy. After modification with SAMs, the surface energy of the material is ideally determined entirely by the monolayer of SAMs. In this case, the polarity (or hydrophilicity/hydrophobicity) differences of the functional groups in the SAM dictate the surface energy of the newly formed surface. Common precursor solutions for perovskite are typically amphiphilic (e.g. solvents like N, N Dimethylformamide and Dimethyl sulfoxide); therefore, to enhance the wettability of perovskites, modifying the substrate to be amphiphilic is a key approach. Recent studies have shown that by adjusting the amphiphilicity of SAM layers, achieving a match with the perovskite precursor solution enables simultaneous optimization of both the perovskite film quality and interface contact [55,94]. Li *et al.* significantly optimized wettability and improved the film formation quality of perovskite by modifying the ITO substrate with two types of SAMs, each having hydrophilic and hydrophobic terminal groups [18]. Zhang *et al.* innovatively designed a bilayer SAM with surface molecules oriented differently (Fig. 6a); the properties of the anchoring groups can be utilized to adjust amphiphilicity [55].

In addition to optimizing physical contact at the interface, SAM-modified substrates can also regulate the crystallization growth process of perovskites. On one hand, SAMs can be used to control grain size; on the other hand, they can reduce pinholes between perovskite grains [19,95]. The ultimate goal is to create a more uniform and high-quality buried perovskite interface. From the perspective of direct regulation, Gu *et al.* found that the carboxyl group in the SAM terminal moiety can serve as a nucleation center for the perovskite layer when negatively charged, thereby further improving the uniformity and smoothness of perovskite crystallization [96]. Zuo *et al.* achieved amino-functionalization of the ZnO surface by using a SAM with amino terminal groups [19], allowing the amino groups to participate in the crystallization process of the perovskite (Fig. 6b). This effectively regulated perovskite growth and significantly reduced pinholes. From the perspective of indirect regulation, Tang *et al.* introduced molecules with bidirectional sulfonic acid groups into the existing SAM, utilizing the Pb defect passivation capability of the sulfonic acid groups to suppress the formation of impurity phases, such as PbI_2_, during perovskite crystallization [97]. Hung *et al.* synthesized a SAM containing a sulfonic acid group, which passivates lead defects, while the pyridine group prevents iodide ion migration. These interactions inhibit nucleation and promote grain growth during the antisolvent step, facilitating a smoother transition from the bottom to the surface during thermal annealing, leading to larger grain sizes and reduced interface trap density [98]. Interestingly, Singh *et al.* found that the SAM-modified substrate exhibited a rougher texture compared to PTAA. This texture induced perovskite growth with a more uniform chemical composition along the vertical interface direction, thereby enhancing operational stability [99].

### Improving interface binding and flexibility

Under stress induced by thermal cycling and externally applied forces, the low interfacial binding strength between functional layers and the inflexible crystal–crystal interface contact accelerate the device degradation process.

A substrate modified with SAMs may exhibit stronger adsorption capabilities on the perovskite surface. This increase in molecular adsorption energy at the microscopic level can explain the enhanced mechanical stability of the interface, such as preventing cracking [21,100]. Dai *et al.* discovered through first-principles calculations that the work of segregation of an iodine-terminated SAM onto the perovskite is twice that of a SAM with a non-halogen terminal group (Fig. 6c). This is due to the bonding between the iodine terminal and the under-coordinated Pb^2+^ exposed on the perovskite surface [21]. The simultaneous formation of chemical bonds by both the anchoring group and the terminal group significantly enhances the interface strength. Dai *et al.* also found that a carbazole-based SAM with an iodine terminal group can increase the interfacial mechanical adhesion by 2.6-fold [101]. Additionally, beyond the aforementioned discussion on chemical adsorption, SAMs may also introduce additional electrostatic interactions, enhancing interfacial binding strength and contact quality. For instance, electrostatic interactions between protonated amine groups and acidic PSS can contribute to improved interface properties [96].

In addition to interface cracking caused by weak bonding, thermal expansion mismatch between different materials due to external temperature changes can also increase interface stress, leading to cracking. The introduction of SAMs, which act as a method to separate the crystal-to-crystal interface, can release thermal stress through the inherent flexibility of organic molecules [20,102]. Park *et al.* evaluated the impact of SAM length, anchored on the SnO_2_ substrate, on its stress-relief capability. The results showed that longer SAMs exhibited better stress-relief performance [102]. Isikgor *et al.* suggested that this stress relief (Fig. 6d) is achieved through the structural freedom inherent in SAMs [20]. It is worth noting that similar stress-relief mechanisms are also applicable in flexible devices. For instance, Dai *et al.* introduced a SAM layer between the SnO_2_ transport layer and the perovskite, significantly enhancing the bending stability of flexible PSCs [103].

### Enhanced durability through physical protection and chemical anchoring

The nature of perovskite as an ionic crystal with a soft lattice structure makes it highly susceptible to ion migration under the influence of an electric field, which can lead to chemical reactions at the interfaces or phase segregation. These processes compromise the chemical stability of the interface. The stability is further reduced when the perovskite has a complex alloy composition and is exposed to external water and oxygen.

A direct approach to addressing unfavorable chemical reactions is to physically separate the reactants by using a barrier layer to block their contact. Forming a dense SAM assembly on the perovskite surface can prevent ion migration and water erosion through steric effects or hydrophobicity, thereby significantly enhancing the stability of the interface [14,104]. Li *et al.* passivated the perovskite surface with carbon dots, followed by modification with a trichloro(3,3,3-trifluoropropyl)silane SAM layer [14]. The hydrophobicity of the -CF_3_ terminal group effectively mitigated the poor interfacial water stability caused by the hydrophilicity of the carbon dots (Fig. 6e). Wang *et al.* designed a self-assembled cocrystal layer at the perovskite/Spiro-OMeTAD interface [104]. The excellent steric hindrance and hydrophobic effects of the large pyrene rings and fluorine atoms inhibited the bidirectional migration of halides and Li^+^, while also suppressing water and oxygen corrosion (Fig. 6f).

Phase segregation results from extensive ion migration, a process that can be inhibited through chemical anchoring methods. The addition of SAMs can enhance the ion migration barrier through interactions with ions on the perovskite surface [23,70,105]. Liu *et al.* demonstrated through first-principles calculations that anchoring an amphiphilic SAM layer (Z907) on the perovskite surface (Fig. 6g) can double the migration barrier for surface halides [105]. Zhang *et al.* found that adjusting the length of the terminal groups in the SAM and employing bidirectional bridging anchoring (Fig. 6h), as opposed to unidirectional anchoring, further optimized phase stability [23]. Chen *et al.* achieved bridged anchoring by incorporating an extra chemically active functional group (methylthio) into the terminal groups [106].

In addition to direct anchoring, SAMs can also suppress ion migration through indirect mechanisms. Localized distortions caused by defects can reduce the activation energy for ion movement. Passivating defects with SAMs is an indirect method to inhibit ion migration [107]. Hydroxyl groups on the substrate are another factor that promotes ion migration. Reacting these hydroxyl groups with SAMs can also hinder the ion migration and aggregation processes [22]. Interestingly, recent studies by Merino *et al.* have shown that unfavorable energy level alignment at the interface, which leads to carrier accumulation, can also contribute to phase segregation [108]. This issue can be effectively addressed using the mixed SAM approach discussed earlier.

## CONCLUSION AND PERSPECTIVE

### Conclusion

In conclusion, this review has demonstrated the multifaceted roles of SAMs in addressing critical challenges facing the large-scale industrialization of perovskite optoelectronic devices. By precisely modifying interfacial properties, SAMs offer a powerful strategy to enhance device performance and stability. From an electronic perspective, SAMs can effectively passivate interfacial defects, optimize energy level alignment and facilitate efficient charge carrier transport. Furthermore, SAMs exert significant influence on the crystal growth process, enabling control over film morphology and suppressing detrimental phase segregation. Mechanically, SAMs can enhance interfacial adhesion, mitigate stress and improve device robustness. Chemically, they act as protective barriers, preventing degradation pathways such as ion migration and moisture ingress.

### Design rules of SAMs

As interlayer materials primarily used for surface and interface modification, SAMs should be designed in a function-oriented manner. First, it is necessary to determine the interlayer location of SAMs. Specifically, based on the target properties of perovskite optoelectronic devices requiring optimization, designers should identify the primary limiting interfaces and decide whether to replace charge transport layer materials or modify interfaces at specific locations. The interlayer location of SAMs directly dictates the types of materials they interact with. For example, the buried interface typically requires anchoring effects on metal oxide surfaces, whereas the upper perovskite interface requires consideration of halogen or hydrogen bonding interactions formed between SAMs and the perovskite surface. Next, leveraging the detailed classification of SAM effects and atomic-scale mechanisms outlined in this review, the specific molecular structures and functional groups of SAMs should be designed. For instance, hydrophobic groups may be introduced to enhance hydrophobicity, while multi-anchoring structures could improve interfacial bridging. Finally, structural modifications and functional group substitutions should be implemented based on fabrication feasibility and SAMs’ inherent stability. Experimental feedback on property adjustments should guide further optimization, such as dynamically adjusting the Lewis acid-base properties of functional groups in response to defect types or refining molecular dipoles to align energy levels.

### Challenges in SAMs research

Despite these significant advancements, current research and development of SAMs face several critical challenges:

Firstly, the ultrathin nature of SAMs presents significant obstacles in direct characterization. Conventional imaging techniques, such as scanning electron microscopy and transmission electron microscopy, cannot directly visualize the morphology of SAM layers. This necessitates the use of indirect methods, including XPS and SFG vibrational spectroscopy, to probe their structure and properties. This limitation hinders the precise design and optimization of SAMs at the atomic scale.

Secondly, as organic materials, SAMs are susceptible to degradation under operational conditions. In PSCs, for example, SAMs in the bottom transport layer are directly exposed to light, potentially affecting their optical and thermal stability. Furthermore, achieving uniform and defect-free SAM formation remains a challenge, as imperfections in the monolayer can significantly impact device performance.

Thirdly, the rational design of SAMs with specific functionalities for diverse applications remains an area of active research. Tailoring SAMs to meet the unique requirements of different device architectures, such as flexible devices, wide-bandgap/narrow-bandgap devices and large-scale devices, necessitates a deeper understanding of interfacial interactions and the development of advanced design principles.

### Strategies and future directions

These challenges may be addressed through emerging technologies or interdisciplinary collaborative research approaches:

Firstly, developing more refined characterization techniques to determine the atomic-scale local geometric environment is essential. An effective approach is to establish a meaningful connection between the characterization data from scanning tunneling microscopy/AFM, and the molecular geometry and electrostatic potential of the molecules. Alternatively, combining various indirect experimental characterization methods with *ab-initio* molecular dynamics simulations could enable cross-validation of molecular morphology under different conditions [12,42].

Secondly, the intrinsic stability of SAMs can be enhanced through rational design of their structure. This can be achieved through the design of multiple active functional groups to facilitate multiple chemical crosslinks between SAMs and between the SAMs and the interface. Both silanetriol and carboxyl groups, serving as anchoring groups in SAMs, possess the capability for intermolecular crosslinking [109]. Incorporating structures or functional groups with inherent thermal and optical stability into SAMs is also a feasible approach. For example, replacing the alkyl linker in SAMs with a conjugated linker can improve intramolecular electron delocalization, thereby enhancing photostability [110]. Additionally, the use of appropriate additives, solvents, multilayer SAMs or co-SAMs [111] may address issues related to operational stability and film uniformity after SAM film formation. Indirectly, the properties of the anchoring substrate can be rationally tuned to improve the anchoring behavior of SAMs, thereby optimizing their uniformity [112].

Thirdly, data-driven approaches [113], combined with multi-objective optimization algorithms, hold promise for addressing the specific functionalities of SAMs required for device synthesis. One feasible approach is to train reliable property prediction models [114] using material big data and apply optimization algorithms to quickly iterate and determine material characteristics, thereby achieving reverse design. Another approach is to use generative model frameworks such as generative adversarial networks, variational autoencoders, transformers and diffusion to generate SAMs with specific properties, followed by further validation and screening. The data sources can include first-principles calculations or automated high-throughput experimental platforms.

In conclusion, achieving a deep integration of computational materials science [115], data science and experimental science, along with a clear exploration of the relationship between atomic-scale mechanisms and macroscopic properties, is the essential path to solving the current challenges faced by SAMs and accelerating the design of high-performance SAMs.

## Supplementary Material

nwaf150_Supplementary_File_for_Review

## References

[1] Lou Y, Zhang S, Gu Z et al. Perovskite single crystals: dimensional control, optoelectronic properties, and applications. Mater Today 2023; 62: 225–50. 10.1016/j.mattod.2022.11.009

[2] Li H, Hua Y, Wang X et al. Highly stable O-tolylbiguanide-CsPbI_3_ quantum dots and light-emitting diodes by synergistic supramolecular passivation. Adv Funct Mater 2024; 34: 2311554. 10.1002/adfm.202311554

[3] Li T, Luo S, Wang X et al. Alternative lone-pair ns^2^-cation-based semiconductors beyond lead halide perovskites for optoelectronic applications. Adv Mater 2021; 33: 2008574. 10.1002/adma.202008574 34060151

[4] NREL . Best Research-Cell Efficiency Chart. https://www.nrel.gov/pv/interactive-cell-efciency.html (December 2024, date last accessed).

[5] Bai W, Xuan T, Zhao H et al. Perovskite light-emitting diodes with an external quantum efficiency exceeding 30%. Adv Mater 2023; 35: 2302283. 10.1002/adma.202302283 37246938

[6] Lee J-W, Seol D-J, Cho A-N et al. High-efficiency perovskite solar cells based on the black polymorph of HC(NH_2_)_2_PbI_3_. Adv Mater 2014; 26: 4991–8. 10.1002/adma.201401137 24923708

[7] Bi D, Tress W, Dar MI et al. Efficient luminescent solar cells based on tailored mixed-cation perovskites. Sci Adv 2016; 2: e1501170. 10.1126/sciadv.1501170 26767196 PMC4705040

[8] Zang L, Zhao C, Hu X et al. Emerging trends in electron transport layer development for stable and efficient perovskite solar cells. Small 2024; 20: 2400807. 10.1002/smll.202400807 38573941

[9] Anoop KM, Ahipa TN. Recent advancements in the hole transporting layers of perovskite solar cells. Sol Energy 2023; 263: 111937. 10.1016/j.solener.2023.111937

[10] Dong L, Qiu S, Feroze S et al. Simplifying contact-layer design for high-throughput printing of flexible perovskite photovoltaics. Energy Environ Sci 2024; 17: 7147–54. 10.1039/D4EE02707H

[11] Tang H, Shen Z, Shen Y et al. Reinforcing self-assembly of hole transport molecules for stable inverted perovskite solar cells. Science 2024; 383: 1236–40. 10.1126/science.adj9602 38484063

[12] Liu S, Li J, Xiao W et al. Buried interface molecular hybrid for inverted perovskite solar cells. Nature 2024; 632: 536–42. 10.1038/s41586-024-07723-3 38925147

[13] Li Z, Sun X, Zheng X et al. Stabilized hole-selective layer for high-performance inverted p-i-n perovskite solar cells. Science 2023; 382: 284–9. 10.1126/science.ade9637 37856581

[14] Li Z, Guo J, Li Z et al. Incorporating self-assembled silane-crosslinked carbon dots into perovskite solar cells to improve efficiency and stability. J Mater Chem A 2020; 8: 5629–37. 10.1039/D0TA00123F

[15] Soliman AIA, Zhang Y, Zhang L et al. Surface reconstruction of perovskites with organosilanes for high performance and highly stable solar cells. Adv Funct Mater 2025; 35: 2412886. 10.1002/adfm.202412886

[16] Wang Q, Chueh C-C, Zhao T et al. Effects of self-assembled monolayer modification of nickel oxide nanoparticles layer on the performance and application of inverted perovskite solar cells. ChemSusChem 2017; 10: 3794–803. 10.1002/cssc.201701262 28881441

[17] Jin H, Debroye E, Keshavarz M et al. It's a trap! On the nature of localised states and charge trapping in lead halide perovskites. Mater Horiz 2020; 7: 397–410. 10.1039/C9MH00500E

[18] Li E, Bi E, Wu Y et al. Synergistic coassembly of highly wettable and uniform hole-extraction monolayers for scaling-up perovskite solar cells. Adv Funct Mater 2020; 30: 1909509. 10.1002/adfm.201909509

[19] Zuo L, Gu Z, Ye T et al. Enhanced photovoltaic performance of CH_3_NH_3_PbI_3_ perovskite solar cells through interfacial engineering using self-assembling monolayer. J Am Chem Soc 2015; 137: 2674–9. 10.1021/ja512518r 25650811

[20] Isikgor FH, Zhumagali S, Merino LVT et al. Molecular engineering of contact interfaces for high-performance perovskite solar cells. Nat Rev Mater 2023; 8: 89–108. 10.1038/s41578-022-00503-3

[21] Dai Z, Yadavalli SK, Chen M et al. Interfacial toughening with self-assembled monolayers enhances perovskite solar cell reliability. Science 2021; 372: 618–22. 10.1126/science.abf5602 33958474

[22] Tumen-Ulzii G, Matsushima T, Klotz D et al. Hysteresis-less and stable perovskite solar cells with a self-assembled monolayer. Commun Mater 2020; 1: 31. 10.1038/s43246-020-0028-z

[23] Zhang Y, Kong T, Liu Y et al. The effect of self-assembled bridging layer on the performance of pure FAPbI_3_-based perovskite solar cells. Adv Funct Mater 2024; 34: 2401391. 10.1002/adfm.202401391

[24] Gedda M, Gkeka D, Nugraha MI et al. High-efficiency perovskite–organic blend light-emitting diodes featuring self-assembled monolayers as hole-injecting interlayers. Adv Energy Mater 2023; 13: 2201396. 10.1002/aenm.202201396

[25] Aydin E, Ugur E, Yildirim BK et al. Enhanced optoelectronic coupling for perovskite/silicon tandem solar cells. Nature 2023; 623: 732–8. 10.1038/s41586-023-06667-4 37769785

[26] Jiang W, Wang D, Shang W et al. Spin-coated and vacuum-processed hole-extracting self-assembled multilayers with H-aggregation for high-performance inverted perovskite solar cells. Angew Chem Int Ed 2024; 63: e202411730. 10.1002/anie.202411730 39044319

[27] Wang X, Li J, Guo R et al. Regulating phase homogeneity by self-assembled molecules for enhanced efficiency and stability of inverted perovskite solar cells. Nat Photon 2024; 18: 1269–75. 10.1038/s41566-024-01531-x

[28] Bigelow WC, Pickett DL, Zisman WA. Oleophobic monolayers: I. Films adsorbed from solution in non-polar liquids. J Colloid Sci 1946; 1: 513–38. 10.1016/0095-8522(46)90059-1

[29] Ulman A . Formation and structure of self-assembled monolayers. Chem Rev 1996; 96: 1533–54. 10.1021/cr9502357 11848802

[30] Sun S . Perspective on the application of self-assembled monolayers in batteries. Acta Phys Chim Sin 2023; 39: 2109022.

[31] Wojciechowski K, Stranks SD, Abate A et al. Heterojunction modification for highly efficient organic–inorganic perovskite solar cells. ACS Nano 2014; 8: 12701–9. 10.1021/nn505723h 25415931

[32] Al-Ashouri A, Magomedov A, Roß M et al. Conformal monolayer contacts with lossless interfaces for perovskite single junction and monolithic tandem solar cells. Energy Environ Sci 2019; 12: 3356–69. 10.1039/C9EE02268F

[33] Magomedov A, Al-Ashouri A, Kasparavičius E et al. Self-assembled hole transporting monolayer for highly efficient perovskite solar cells. Adv Energy Mater 2018; 8: 1801892. 10.1002/aenm.201801892

[34] Jiang W, Li F, Li M et al. π-Expanded carbazoles as hole-selective self-assembled monolayers for high-performance perovskite solar cells. Angew Chem 2022; 134: e202213560. 10.1002/ange.202213560 36300589

[35] He R, Wang W, Yi Z et al. Improving interface quality for 1-cm^2^ all-perovskite tandem solar cells. Nature 2023; 618: 80–6. 10.1038/s41586-023-05992-y 36990110

[36] Yalcin E, Can M, Rodriguez-Seco C et al. Semiconductor self-assembled monolayers as selective contacts for efficient PiN perovskite solar cells. Energy Environ Sci 2019; 12: 230–7. 10.1039/C8EE01831F

[37] Ullah A, Park KH, Lee Y et al. Versatile hole selective molecules containing a series of heteroatoms as self-assembled monolayers for efficient p-i-n perovskite and organic solar cells. Adv Funct Mater 2022; 32: 2208793. 10.1002/adfm.202208793

[38] Tan Q, Li Z, Luo G et al. Inverted perovskite solar cells using dimethylacridine-based dopants. Nature 2023; 620: 545–51. 10.1038/s41586-023-06207-0 37224876

[39] Li L, Wu Y, Li E et al. Self-assembled naphthalimide derivatives as an efficient and low-cost electron extraction layer for n-i-p perovskite solar cells. Chem Commun 2019; 55: 13239–42. 10.1039/C9CC06345E 31620721

[40] Fürer SO, Rietwyk KJ, Pulvirenti F et al. Naphthalene-imide self-assembled monolayers as a surface modification of ITO for improved thermal stability of perovskite solar cells. ACS Appl Energy Mater 2023; 6: 667–77. 10.1021/acsaem.2c02735

[41] Chen Z, Li Y, Liu Z et al. Reconfiguration toward self-assembled monolayer passivation for high-performance perovskite solar cells. Adv Energy Mater 2023; 13: 2202799. 10.1002/aenm.202202799

[42] Park SM, Wei M, Lempesis N et al. Low-loss contacts on textured substrates for inverted perovskite solar cells. Nature 2023; 624: 289–94. 10.1038/s41586-023-06745-7 37871614

[43] Kim G, Kim H, Kim M et al. Enhancing surface modification and carrier extraction in inverted perovskite solar cells via self-assembled monolayers. Nanomaterials 2024; 14: 214. 10.3390/nano14020214 38276732 PMC10821478

[44] Arkan E, Yigit Arkan MZ, Unal M et al. Performance enhancement of inverted perovskite solar cells through interface engineering by TPD based bidentate self-assembled monolayers. Opt Mater 2020; 105: 109910. 10.1016/j.optmat.2020.109910

[45] Wang Y, Liao Q, Chen J et al. Teaching an old anchoring group new tricks: enabling low-cost, eco-friendly hole-transporting materials for efficient and stable perovskite solar cells. J Am Chem Soc 2020; 142: 16632–43. 10.1021/jacs.0c06373 32852200

[46] Levine I, Al-Ashouri A, Musiienko A et al. Charge transfer rates and electron trapping at buried interfaces of perovskite solar cells. Joule 2021; 5: 2915–33. 10.1016/j.joule.2021.07.016

[47] Li E, Liu C, Lin H et al. Bonding strength regulates anchoring-based self-assembly monolayers for efficient and stable perovskite solar cells. Adv Funct Mater 2021; 31: 2103847. 10.1002/adfm.202103847

[48] Guo H, Liu C, Hu H et al. Neglected acidity pitfall: boric acid-anchoring hole-selective contact for perovskite solar cells. Natl Sci Rev 2023; 10: nwad057. 10.1093/nsr/nwad057 37274941 PMC10237332

[49] Kim SY, Kang H, Chang K et al. Case studies on structure–property relations in Perovskite light-emitting diodes via interfacial engineering with self-assembled monolayers. ACS Appl Mater Interfaces 2021; 13: 31236–47. 10.1021/acsami.1c03797 34170098

[50] Cheng H, Li Y, Zhang M et al. Self-assembled ionic liquid for highly efficient electron transport layer-free perovskite solar cells. ChemSusChem 2020; 13: 2779–85. 10.1002/cssc.202000342 32129546

[51] Chen Y, Li B, Zhong W et al. Effect of head groups in self-assembled monolayer passivation on properties of InSnZnO thin-film transistors. IEEE Trans Electron Devices 2022; 69: 160–5. 10.1109/TED.2021.3126568

[52] Kim SY, Cho SJ, Byeon SE et al. Self-assembled monolayers as interface engineering nanomaterials in perovskite solar cells. Adv Energy Mater 2020; 10: 2002606. 10.1002/aenm.202002606

[53] Suo J, Yang B, Bogachuk D et al. The dual use of SAM molecules for efficient and stable perovskite solar cells. Adv Energy Mater 2025; 15: 2400205. 10.1002/aenm.202400205

[54] Wang X, Faizan M, Zhou K et al. Role of self-assembled molecules’ anchoring groups for surface defect passivation and dipole modulation in inverted perovskite solar cells. Chin Phys B 2024; 33: 107303. 10.1088/1674-1056/ad711f

[55] Zhang S, Ye F, Wang X et al. Minimizing buried interfacial defects for efficient inverted perovskite solar cells. Science 2023; 380: 404–9. 10.1126/science.adg3755 37104579

[56] Li M, Liu M, Qi F et al. Self-assembled monolayers for interfacial engineering in solution-processed thin-film electronic devices: design, fabrication, and applications. Chem Rev 2024; 124: 2138–204. 10.1021/acs.chemrev.3c00396 38421811

[57] Wang J, Gadenne V, Patrone L et al. Self-assembled monolayers of push–pull chromophores as active layers and their applications. Molecules 2024; 29: 559. 10.3390/molecules29030559 38338304 PMC10856137

[58] Otsubo T, Aso Y, Takimiya K. Functional oligothiophenes as advanced molecular electronic materials. J Mater Chem 2002; 12: 2565–75. 10.1039/b203780g

[59] Choi B, Rhee J, Lee HH. Tailoring of self-assembled monolayer for polymer light-emitting diodes. Appl Phys Lett 2001; 79: 2109–11. 10.1063/1.1398327

[60] Hatton RA, Day SR, Chesters MA et al. Organic electroluminescent devices: enhanced carrier injection using an organosilane self assembled monolayer (SAM) derivatized ITO electrode. Thin Solid Films 2001; 394: 291–6. 10.1016/S0040-6090(01)01191-9

[61] Khodabakhsh S, Sanderson BM, Nelson J et al. Using self-assembling dipole molecules to improve charge collection in molecular solar cells. Adv Funct Mater 2006; 16: 95–100. 10.1002/adfm.200500207

[62] Senadeera GKR, Kitamura T, Wada Y et al. Deposition of polyaniline via molecular self-assembly on TiO_2_ and its uses as a sensitiser in solid-state solar cells. J Photochem Photobiol A: Chem 2004; 164: 61–6. 10.1016/j.jphotochem.2003.12.026

[63] Lin S-C, Lee Y-L, Chang C-H et al. Quantum-dot-sensitized solar cells: assembly of CdS-quantum-dots coupling techniques of self-assembled monolayer and chemical bath deposition. Appl Phys Lett 2007; 90: 143517. 10.1063/1.2721373

[64] Li Y, Xie H, Lim EL et al. Recent progress of critical interface engineering for highly efficient and stable perovskite solar cells. Adv Energy Mater 2022; 12: 2102730. 10.1002/aenm.202102730

[65] Li L, Wang Y, Wang X et al. Flexible all-perovskite tandem solar cells approaching 25% efficiency with molecule-bridged hole-selective contact. Nat Energy 2022; 7: 708–17. 10.1038/s41560-022-01045-2

[66] Krogmeier B, Staub F, Grabowski D et al. Quantitative analysis of the transient photoluminescence of CH_3_NH_3_PbI_3_/PC_61_BM heterojunctions by numerical simulations. Sustain Energy Fuels 2018; 2: 1027–34. 10.1039/C7SE00603A

[67] Al-Ashouri A, Köhnen E, Li B et al. Monolithic perovskite/silicon tandem solar cell with >29% efficiency by enhanced hole extraction. Science 2020; 370: 1300–9. 33303611 10.1126/science.abd4016

[68] Wang Y-K, Jia F, Li X et al. Self-assembled monolayer–based blue perovskite LEDs. Sci Adv 2023; 9: eadh2140. 10.1126/sciadv.adh2140 37683007 PMC10491221

[69] Chen B, Guo R, He Z et al. Self-assembled monolayers as hole transport layers for efficient thermally evaporated blue perovskite light-emitting diodes. Chem Eng J 2023; 476: 146476. 10.1016/j.cej.2023.146476

[70] Zhang S, Li M, Zeng H et al. Grain boundary and buried interface suturing enabled by fullerene derivatives for high-performance perovskite solar module. ACS Energy Lett 2022; 7: 3958–66. 10.1021/acsenergylett.2c01854

[71] Sekimoto T, Yamamoto T, Takeno F et al. Perovskite solar cell using isonicotinic acid as a gap-filling self-assembled monolayer with high photovoltaic performance and light stability. ACS Appl Mater Interfaces 2023; 15: 33581–92. 10.1021/acsami.3c05215 37417321

[72] Jiang W, Liu M, Li Y et al. Rational molecular design of multifunctional self-assembled monolayers for efficient hole selection and buried interface passivation in inverted perovskite solar cells. Chem Sci 2024; 15: 2778–85. 10.1039/D3SC05485C 38404377 PMC10882494

[73] Cho H, Jeong S-H, Park M-H et al. Overcoming the electroluminescence efficiency limitations of perovskite light-emitting diodes. Science 2015; 350: 1222–5. 10.1126/science.aad1818 26785482

[74] Zhao Y, Tan H, Yuan H et al. Perovskite seeding growth of formamidinium-lead-iodide-based perovskites for efficient and stable solar cells. Nat Commun 2018; 9: 1607. 10.1038/s41467-018-04029-7 29686304 PMC5913260

[75] Hou M, Zhang H, Wang Z et al. Enhancing efficiency and stability of Perovskite solar cells via a self-assembled dopamine interfacial layer. ACS Appl Mater Interfaces 2018; 10: 30607–13. 10.1021/acsami.8b10332 30118201

[76] Ju S-Y, Lee WI, Kim H-S. Enhanced phase stability of compressive strain-induced perovskite crystals. ACS Appl Mater Interfaces 2022; 14: 39996–40004. 10.1021/acsami.2c10450 36008374

[77] Zhang J, Yang J, Dai R et al. Elimination of interfacial lattice mismatch and detrimental reaction by self-assembled layer dual-passivation for efficient and stable inverted perovskite solar cells. Adv Energy Mater 2022; 12: 2103674. 10.1002/aenm.202103674

[78] Cao Q, Wang T, Pu X et al. Co-self-assembled monolayers modified NiO for stable inverted perovskite solar cells. Adv Mater 2024; 36: 2311970. 10.1002/adma.202311970 38198824

[79] Lv X, Xu Y-L, Sun S-Q et al. High-performance all-inorganic perovskite light-emitting diodes enabled by a self-assembled molecule additive via defect passivation and strain relaxation. J Alloys Compd 2023; 969: 172459. 10.1016/j.jallcom.2023.172459

[80] Dong Q, Chen M, Liu Y et al. Flexible perovskite solar cells with simultaneously improved efficiency, operational stability, and mechanical reliability. Joule 2021; 5: 1587–601. 10.1016/j.joule.2021.04.014

[81] Yang IS, Dai Z, Ranka A et al. Simultaneous enhancement of efficiency and operational-stability of mesoscopic perovskite solar cells via interfacial toughening. Adv Mater 2024; 36: 2308819. 10.1002/adma.202308819 37832157

[82] Frost JM, Butler KT, Brivio F et al. Atomistic origins of high-performance in hybrid halide perovskite solar cells. Nano Lett 2014; 14: 2584–90. 10.1021/nl500390f 24684284 PMC4022647

[83] Boyd CC, Shallcross RC, Moot T et al. Overcoming redox reactions at perovskite-nickel oxide interfaces to boost voltages in perovskite solar cells. Joule 2020; 4: 1759–75. 10.1016/j.joule.2020.06.004

[84] Han J, Kwon H, Kim E et al. Interfacial engineering of a ZnO electron transporting layer using self-assembled monolayers for high performance and stable perovskite solar cells. J Mater Chem A 2020; 8: 2105–13. 10.1039/C9TA12750J

[85] Li B, Zhang C, Gao D et al. Suppressing oxidation at perovskite–NiO interface for efficient and stable tin perovskite solar cells. Adv Mater 2024; 36: 2309768. 10.1002/adma.202309768 37971969

[86] Lin J, Wang Y, Khaleed A et al. Dual surface modifications of NiO_x_/perovskite interface for enhancement of device stability. ACS Appl Mater Interfaces 2023; 15: 24437–47. 10.1021/acsami.3c02156 37150934

[87] Mussakhanuly N, Choi E, Chin RL *et al*. Multifunctional surface treatment against imperfections and halide segregation in wide-band gap perovskite solar cells. ACS Appl Mater Interfaces 2024; 16: 7961–72. 10.1021/acsami.3c12616 38290432

[88] Fei C, Li N, Wang M et al. Lead-chelating hole-transport layers for efficient and stable perovskite minimodules. Science 2023; 380: 823–9. 10.1126/science.ade9463 37228201

[89] Hung C-M, Mai C-L, Wu C-C et al. Self-assembled monolayers of Bi-functionalized porphyrins: a novel class of hole-layer-coordinating perovskites and indium tin oxide in inverted solar cells. Angew Chem Int Ed 2023; 62: e202309831. 10.1002/anie.202309831 37594921

[90] Zhu T, Olthof S, Pauporté T. Titanium dioxide surface energy levels tuning by self-assembled monolayers. Appl Phys Lett 2022; 121: 141602. 10.1063/5.0107202

[91] Wang Y, Akel S, Klingebiel B et al. Hole transporting bilayers for efficient micrometer-thick perovskite solar cells. Adv Energy Mater 2024; 14: 2302614. 10.1002/aenm.202302614

[92] Zhang Z, Zhu R, Tang Y et al. Anchoring charge selective self-assembled monolayers for tin–lead perovskite solar cells. Adv Mater 2024; 36: 2312264. 10.1002/adma.202312264 38281081

[93] Bi C, Wang Q, Shao Y et al. Non-wetting surface-driven high-aspect-ratio crystalline grain growth for efficient hybrid perovskite solar cells. Nat Commun 2015; 6: 7747. 10.1038/ncomms8747 26190275 PMC4518278

[94] Liu S-C, Lin H-Y, Hsu S-E et al. Highly reproducible self-assembled monolayer based perovskite solar cells via amphiphilic polyelectrolyte. J Mater Chem A 2024; 12: 2856–66. 10.1039/D3TA04512A

[95] Lu H, Zhuang J, Ma Z et al. γ-MPTS-SAM modified meso-TiO_2_ surface to enhance performance in perovskite solar cell. Mater Sci Semicond Process 2019; 97: 21–8. 10.1016/j.mssp.2019.02.018

[96] Gu Z, Zuo L, Larsen-Olsen TT et al. Interfacial engineering of self-assembled monolayer modified semi-roll-to-roll planar heterojunction perovskite solar cells on flexible substrates. J Mater Chem A 2015; 3: 24254–60. 10.1039/C5TA07008B

[97] Tang Y, Zhang Z, Liu H et al. Multifunctional action site strategy of a buried interface for high-performance perovskite solar cells. ACS Photon 2024; 11: 4916–22. 10.1021/acsphotonics.4c01426

[98] Hung C-M, Wu C-C, Yang Y-H et al. Repairing interfacial defects in self-assembled monolayers for high-efficiency perovskite solar cells and organic photovoltaics through the SAM@Pseudo-planar monolayer strategy. Adv Sci 2024; 11: 2404725. 10.1002/advs.202404725 PMC11423173 39078745

[99] Singh S, Siliavka E, Löffler M et al. Impact of buried interface texture on compositional stratification and ion migration in perovskite solar cells. Adv Funct Mater 2024; 34: 2402655. 10.1002/adfm.202402655

[100] Almasabi K, Zheng X, Turedi B et al. Hole-transporting self-assembled monolayer enables efficient single-crystal perovskite solar cells with enhanced stability. ACS Energy Lett 2023; 8: 950–6. 10.1021/acsenergylett.2c02333

[101] Dai Z, You S, Chakraborty D et al. Connecting interfacial mechanical adhesion, efficiency, and operational stability in high performance inverted perovskite solar cells. ACS Energy Lett 2024; 9: 1880–7. 10.1021/acsenergylett.4c00510

[102] Park D-A, Zhang C, Park N-G. Strain-less perovskite film engineered by interfacial molecule for stable perovskite solar cells. ACS Energy Lett 2024; 9: 2428–35. 10.1021/acsenergylett.4c00656

[103] Dai Z, Li S, Liu X et al. Dual-interface-reinforced flexible perovskite solar cells for enhanced performance and mechanical reliability. Adv Mater 2022; 34: 2205301. 10.1002/adma.202205301 36148590

[104] Wang X, Zhong Y, Luo X et al. Elimination of charge accumulation by a self-assembled cocrystal interlayer for efficient and stable perovskite solar cells. Energy Environ Sci 2024; 17: 569–79. 10.1039/D3EE03550F

[105] Liu L, Yang Y, Du M et al. Self-assembled amphiphilic monolayer for efficient and stable wide-bandgap perovskite solar cells. Adv Energy Mater 2023; 13: 2202802. 10.1002/aenm.202202802

[106] Chen C-H, Liu G-W, Chen X et al. Methylthio substituent in SAM constructing regulatory bridge with photovoltaic perovskites. Angew Chem Int Ed 2025; 64: e202419375. 10.1002/anie.202419375 39618342

[107] Deng X, Qi F, Li F et al. Co-assembled monolayers as hole-selective contact for high-performance inverted perovskite solar cells with optimized recombination loss and long-term stability. Angew Chem 2022; 134: e202203088. 10.1002/ange.202203088 35560775

[108] Torres Merino LV, Petoukhoff CE, Matiash O et al. Impact of the valence band energy alignment at the hole-collecting interface on the photostability of wide band-gap perovskite solar cells. Joule 2024; 8: 2585–606. 10.1016/j.joule.2024.06.017

[109] Bai Y, Dong Q, Shao Y et al. Enhancing stability and efficiency of perovskite solar cells with crosslinkable silane-functionalized and doped fullerene. Nat Commun 2016; 7: 12806. 10.1038/ncomms12806 27703136 PMC5059465

[110] Zhang S, Wu R, Mu C et al. Conjugated self-assembled monolayer as stable hole-selective contact for inverted perovskite solar cells. ACS Mater Lett 2022; 4: 1976–83. 10.1021/acsmaterialslett.2c00799

[111] Liu W, Zang Y, Tu Y et al. Reconstruction of hole transport layer via Co-self-assembled molecules for high-performance inverted perovskite solar cells. Small 2025; 21: 2408314. 10.1002/smll.202408314 39552011

[112] Jin Y, Feng H, Li Y et al. Recrystallizing sputtered NiO for improved hole extraction in perovskite/silicon tandem solar cells. Adv Energy Mater 2025; 15: 2403911. 10.1002/aenm.202403911

[113] Zhang Q, Wang H, Zhao Q et al. Machine-learning-assisted design of buried-interface engineering materials for high-efficiency and stable perovskite solar cells. ACS Energy Lett 2024; 9: 5924–34. 10.1021/acsenergylett.4c02610

[114] Zhao R, Xing B, Mu H et al. Evaluation of performance of machine learning methods in mining structure–property data of halide perovskite materials. Chin Phys B 2022; 31: 056302. 10.1088/1674-1056/ac5d2d

[115] Zhao X-G, Zhou K, Xing B et al. JAMIP: an artificial-intelligence aided data-driven infrastructure for computational materials informatics. Sci Bull 2021; 66: 1973–85. 10.1016/j.scib.2021.06.011 36654167

