# Supplementary material for: Role of self-assembled molecules in halide perovskite optoelectronics: an atomic-scale perspective
Source: Natl Sci Rev. 2025 Apr 24;12(5):nwaf150. doi: 10.1093/nsr/nwaf150 (PMC12096312; doi:10.1093/nsr/nwaf150)
Supplement: nwaf150_Supplementary_File_for_Review [file nwaf150_supplementary_file_for_review.pdf]

Supplemental Material for

Role of Self-Assembled Molecules in Halide Perovskite Optoelectronics: An Atomic-Scale Perspective

Xiaoyu Wang[1,†], Xue Wang[2,†], Xinjiang Wang[2], Hanming Li[2], Yuhao Fu[1,*] and Lijun Zhang[2,*]

[1] *State Key Laboratory of Superhard Materials, College of Physics, Jilin University, Changchun 130012, China*

[2] *State Key Laboratory of Integrated Optoelectronics, Key Laboratory of Automobile Materials of MOE, College of Materials Science and Engineering, Jilin University, Changchun 130012, China*

[*]Corresponding author.

E-mail address: fuyuhaoy@gmail.com (Y. Fu), lijun_zhang@jlu.edu.cn (L. Zhang)

[†]These authors contribute equally: Xiaoyu Wang and Xue Wang.

Table S1: A summary of research published between 2018 and 2024 on the optimization of perovskite optoelectronic devices using self-assembled molecules, focusing on device efficiency and stability. PSC: perovskite solar cell, PeLED: perovskite light-emitting diodes, Si/P TSC: silicon-perovskite tandem solar cell, 2DRP-PSC: two-dimensional Ruddlesden–Popper perovskite solar cell, P/P TSC: all perovskite tandem solar cell, MPP: maximum power point, PCE: power conversion efficiency, RH: relative humidity.

SAM	Device type	Efficiency	Condition	Time (h)	Stability (%)	ref
Me-4PACz	PSC	PCE 25.25 %	unencapsulated/nitrogen/dark/25 ℃	1600	90	[1]
Me-4PACz	PSC	PCE 25.25 %	unencapsulated/nitrogen/dark/65 ℃	1600	80	[1]
Me-4PACz	PSC	PCE 25.25 %	MPP/encapsulated/nitrogen/55 ℃	800	80	[1]
MeS−CbzPh	PSC	PCE 26.01 %	MPP/unencapsulated/nitrogen	1000	93.3	[2]

MeO−CbzPh	PSC	PCE 25.16 %	MPP/unencapsulated/nitrogen	1000	85.5	[2]
CbzPh	PSC	PCE 24.36 %	MPP/unencapsulated/nitrogen	1000	77.8	[2]
TTP-COOMA	PSC	PCE 24.22 %	MPP/encapsulated/air/25 ℃	1000	93.6	[3]
TTP-COOMA	PSC	PCE 24.22 %	MPP/encapsulated/air/45 ℃	1000	88.7	[3]
TTP-COOMA	PSC	PCE 24.22 %	MPP/encapsulated/air/65 ℃	1000	82.4	[3]
TTP-COOMA	PSC	PCE 24.22 %	MPP/encapsulated/air/85 ℃	1000	78.8	[3]
Br-2PET	PeLED	EQE 6.56 % (457 nm)	100 cd m^{-2}	0.67	50	[4]
C-2PACz	PeLED	EQE 10.41 % (484 nm)	-	0.335	50	[5]
PAPzO	PSC	PCE 26.04 %	MPP/unencapsulated/nitrogen/25 ℃	1200	91.24	[6]
PAPzO	PSC	PCE 26.04 %	unencapsulated/air/65 ℃	1400	92.93	[6]
TPAT-CA	Tin-PSC	PCE 8.1 %	unencapsulated/nitrogen/dark	3000	90	[7]
TPAT-MN	Tin-PSC	PCE 6.1 %	-	-	-	[7]
TPA-MN	Tin-PSC	PCE 5.0 %	-	-	-	[7]
TPA-CA	Tin-PSC	PCE 4.2 %	-	-	-	[7]
KF	PSC	PCE 25.17 %	unencapsulated/65 ℃/50 % RH	645	80	[8]
KF	PSC	PCE 25.17 %	MPP/encapsulated/air	420	93	[8]
tBu-4PACz	PSC	PCE 26.21 %	MPP/unencapsulated/nitrogen	500	94.7	[9]
2PICz	PSC	PCE 25.51 %	unencapsulated/nitrogen/25 ℃	3000	98.6	[10]
2PICz	PSC	PCE 25.51 %	unencapsulated/nitrogen/65 ℃	2000	87.5	[10]
2PICz	PSC	PCE 25.51 %	MPP/unencapsulated/nitrogen	650	80	[10]
CbzNaphPPA	PSC	PCE 26.07 %	MPP/unencapsulated/nitrogen/65 ℃	1200	94	[11]
Me-4PACz/PA	Si/P TSC	PCE 30.22 %	-	-	-	[12]
DMAcPA/4NPBA	PSC	PCE 25.29 %	MPP/encapsulated/nitrogen/25 ℃	500	99	[13]
Me-4PACz/2PACz/HEA	PSC	PCE 25.71 %	MPP/unencapsulated/nitrogen	1000	91.8	[14]
PA	PSC	PCE 23.3 %	nitrogen/85 ℃	500	65	[15]
4PAPyr	PSC	PCE 22.2 %	MPP/65 ℃	16	77	[16]

MoO3/2PACz	PeLED	EQE 18.2 % (515 nm)	200 cd m^{-2}	0.183	50	[17]
Z907	Si/P TSC	PCE 32.07 %	unencapsulated/air/25 ℃/30~40 % RH	1000	97.2	[18]
PANI/MeO-2PACz	PSC	PCE 25.59 %	unencapsulated/nitrogen/25 ℃/5~10 % RH	1200	97.3	[19]
PANI/MeO-2PACz	PSC	PCE 25.59 %	MPP/unencapsulated/nitrogen/65 ℃	500	88	[19]
2PACz-Br	2DRP-PSC	PCE 19.13 %	unencapsulated/nitrogen/25 ℃/50 % RH	300	85	[20]
Py2	PSC	PCE 25.2 %	MPP/encapsulated/air/35 ℃/55 % RH	1000	90	[21]
Py2	PSC	PCE 25.2 %	MPP/encapsulated/air/55 ℃/55 % RH	830	90	[21]
DMPU/Me-4PACz	PSC	PCE 22.4 %	MPP/encapsulated/nitrogen	490	80	[22]
PEACl/MeO-2PACz	PSC	PCE 24.11 %	MPP/unencapsulated/nitrogen/45 ℃	1500	97	[23]
PEACl/MeO-2PACz	PSC	PCE 24.11 %	unencapsulated/air/25~45 ℃/10 % RH	4000	94	[23]
PEACl/MeO-2PACz	PSC	PCE 24.11 %	unencapsulated/nitrogen/75 ℃	500	85	[23]
Br-2PACz	All-inorganic PSC	PCE 19.34 %	unencapsulated/air/30~35 % RH	960	80	[24]
FPTMS/MeO-4PACz	PSC	PCE 23.0 %	MPP/encapsulated/air/35 ℃/50 % RH	1725	85	[25]
Ph-4PACz	PSC	PCE 25.2 % (1 cm^2)	MPP/encapsulated/85 % RH	600	100	[26]
Ph-4PACz	PSC	PCE 25.2 % (1 cm^2)	encapsulated/85 ℃	1000	90	[26]
AlOx/F-PTABr/MeO-2PACz	PSC	PCE 21.53 %	unencapsulated/nitrogen/dark/25 ℃	3150	93.5	[27]
AlOx/F-PTABr/MeO-2PACz	PSC	PCE 21.53 %	encapsulated/air/85 ℃/20~30 % RH	600	90	[27]
MeO-2PACz	PeLED	EQE 21.5 % (513 nm)	1000 cd m^{-2}	2.6	50	[28]
MeO-2PACz	PeLED	EQE 21.5 % (513 nm)	1000 cd m^{-2}	14.5	50	[28]
2PACz	PeLED	EQE 15.2 % (491 nm)	~80 cd m^{-2}	0.044	50	[29]
PyCA-3F/2PACz	PSC	PCE 24.68 %	MPP/encapsulated/air/25 ℃/60 ± 10 % RH	1000	90	[30]
PyCA-3F/2PACz	PSC	PCE 24.68 %	unencapsulated/nitrogen/65 ℃	650	92	[30]
TP-MN	Tin-PSC	PCE 7.7 %	MPP/unencapsulated/air	7	85	[31]
TP-CA	Tin-PSC	PCE 4.8 %	-	-	-	[31]
TPT-MN	Tin-PSC	PCE 4.6 %	-	-	-	[31]
Me-4PACz	PeLED	EQE 4.33 % (458 nm)	-	0.16	50	[32]

XS8	PeLED	EQE 12.0 % (519 nm)	-	-	-	[33]
XS9	PeLED	EQE 11.2 % (519 nm)	-	-	-	[33]
XS10	PeLED	EQE 16.6 % (519 nm)	-	-	-	[33]
XS8	PSC	PCE 16.11 %	-	-	-	[33]
XS9	PSC	PCE 15.77 %	-	-	-	[33]
XS10	PSC	PCE 20.28 %	-	-	-	[33]
Glycine/2PACz	PSC	PCE 23.46 %	unencapsulated/nitrogen	1680	91.8	[34]
Glycine/2PACz	PSC	PCE 23.46 %	unencapsulated/nitrogen/65 ℃	590	88	[34]
S-2PACz	PSC	PCE 18.65 %	unencapsulated/nitrogen	720	85	[35]
C-2PACz	PSC	PCE 18.16 %	unencapsulated/nitrogen	720	80	[35]
FAPA	PSC	PCE 17.8 %	unencapsulated/air	1200	90	[36]
FAPA	PSC	PCE 17.8 %	unencapsulated/1 sun/65 ℃	200	85	[36]
X13	PSC	PCE 19.2 %	encapsulation/nitrogen	720	85	[37]
MPS	PeLED	EQE 10.4 % (489 nm)	-	0.13	50	[38]
CBZ-B-PY/CNph	PSC	PCE 22.89 %	-	-	-	[39]
DPA-B-PY/CNph	PSC	PCE 23.25 %	-	-	-	[39]
POZ-B-PY/CNph	PSC	PCE 23.67 %	-	-	-	[39]
POZ-PY/CNph	PSC	PCE 23.72 %	-	-	-	[39]
POZ-T-PY/CNph	PSC	PCE 23.93 %	-	-	-	[39]
POZ-BT-PY/CNph	PSC	PCE 24.45 %	air/25 ± 5 % RH	720	93	[39]
POZ-BT-PY/CNph	PSC	PCE 24.45 %	nitrogen/65 ℃	720	87	[39]
A-4PADCB	f-PSC	PCE 25.05 %	MPP/unencapsulated/air	400	87	[40]
Me-PhpPACz	PSC	PCE 26.17 %	MPP/unencapsulated/nitrogen/50 ± 10 ℃	3100	98.7	[41]
4PADCB	PSC	PCE 24.8 %	unencapsulated/nitrogen/85 ℃	1000	80	[42]
4PADCB	PSC	PCE 24.8 %	MPP/unencapsulated/air	500	80	[42]
Me-4PACz	All-inorganic PSC	PCE 20.21 %	unencapsulated/air/22~25 ℃/30 % RH	200	83	[43]

GM-4PACz	PSC	PCE 25.52 %	unencapsulated/air/25 °C/30 % RH	2000	93.29	[44]
GM-4PACz	PSC	PCE 25.52 %	unencapsulated/nitrogen/85 °C	500	88.13	[44]
NA/Me-4PACz	PSC	PCE 26.54 %	encapsulated/85 °C/85 % RH	500	97.4	[45]
NA/Me-4PACz	PSC	PCE 26.54 %	MPP/encapsulated/air/65 °C	2400	96.1	[45]
2PACz	All-inorganic PSC	PCE 18.89 %	unencapsulated/nitrogen	1000	86	[46]
2PACz	All-inorganic PSC	PCE 18.89 %	MPP/unencapsulated/nitrogen	400	89	[46]
Me-4PACz/MeO-PhPACz	PSC	PCE 22.63 %	MPP/unencapsulated/nitrogen	1000	100	[47]
Me-4PACz/MeO-PhPACz	PSC	PCE 22.63 %	encapsulated/85 °C/85 % RH	900	90	[47]
2-MeIM/MeO-2PACz	PSC	PCE 24.38 %	nitrogen/25 °C	2200	78	[48]
2-MeBIM/MeO-2PACz	PSC	PCE 24.09 %	nitrogen/25 °C	2200	75	[48]
2-MeIM/MeO-2PACz	PSC	PCE 24.38 %	nitrogen/70 °C	1080	86	[48]
2-MeBIM/MeO-2PACz	PSC	PCE 24.09 %	nitrogen/70 °C	1080	84	[48]
I-2PACz	PSC ($FAPbBr_3$)	PCE 11.14 %	MPP/encapsulated/45 °C	1000	93	[49]
I-2PACz	PSC ($FAPbBr_3$)	PCE 11.14 %	unencapsulated/air/25 °C/25 % RH	1750	92	[49]
I-2PACz	PSC ($FAPbBr_3$)	PCE 11.14 %	encapsulated/85 °C	1100	86	[49]
Bepo-SAM	PSC	PCE 25.16 %	MPP/encapsulated/40 °C	1000	90	[50]
CEPA	Tin-PSC	PCE 10.65 %	unencapsulated/nitrogen	850	81	[51]
CEPA	Tin-PSC	PCE 10.65 %	unencapsulated/nitrogen/1 sun	240	80	[51]
2PACz	2DRP-PSC	PCE 18.85 %	encapsulated/85 °C/85 % RH	1000	93.6	[52]
2PACz	2DRP-PSC	PCE 18.85 %	MPP/encapsulated/air/65 °C/50 % RH	1000	97.3	[52]
DMPS	PSC	PCE 24.0 %	unencapsulated/dark/28 ± 3 °C/25 ± 5 % RH	2300	90	[53]
DMSS	PSC	PCE 23.1 %	-	-	-	[53]
MESNA	PSC	PCE 20.6 %	-	-	-	[53]
IDCz-3	PSC	PCE 25.15 %	unencapsulated/nitrogen/dark	1800	100	[54]
IDCz-1	PSC	PCE 20.97 %	unencapsulated/nitrogen/dark	1800	76	[54]
IDCz-2	PSC	PCE 23.11 %	unencapsulated/nitrogen/dark	1800	89	[54]

4-PCA	PSC	PCE 23.77 %	unencapsulated/air/20~30 % RH	1000	92.6	[55]
4-PCA	PSC	PCE 23.77 %	unencapsulated/nitrogen/dark/65 ℃	500	81.3	[55]
3-TA	PSC	PCE 22.64 %	-	-	-	[55]
BA	PSC	PCE 22.24 %	-	-	-	[55]
–C3–Cl SAM	PSC	PCE 24.3 %	-	-	-	[56]
–C3≡N SAM	PSC	PCE 25.1 %	MPP/encapsulated/25 ℃/20~40 % RH	1000	97	[56]
MPA-2FBT-BA	PSC	PCE 20.32 %	unencapsulated/nitrogen	2784	93	[57]
MPA-BT-BA	PSC	PCE 19.29 %	unencapsulated/nitrogen	2784	87	[57]
MPA-2FBT-BA	PSC	PCE 20.32 %	unencapsulated/air/30 ± 5 % RH	1008	91	[57]
MPA-BT-BA	PSC	PCE 19.29 %	unencapsulated/air/30 ± 5 % RH	1008	89	[57]
ThPCyAc	PSC	PCE 23.16 %	unencapsulated/air/25 ℃/15 % RH	360	93	[58]
ThPCyAc	PSC	PCE 23.16 %	unencapsulated/80 ℃/35 % RH	120	80	[58]
TBT-BA	PSC	PCE 24.5 %	unencapsulated/nitrogen/60 ℃	2635	88.7	[59]
TBT-FBA	PSC	PCE 24.0 %	unencapsulated/nitrogen/60 ℃	2635	82.4	[59]
TBT-DBA	PSC	PCE 23.1 %	unencapsulated/nitrogen/60 ℃	2635	83.2	[59]
TPATC	PSC	PCE 20.58 %	encapsulated/air/63 ℃/1 sun	1000	92	[60]
2PACz/Me-4PACz	PSC	PCE 19.31 %	MPP/unencapsulated/nitrogen	260	70	[61]
Me-4PACz	PSC	PCE 23.29 %	unencapsulated/nitrogen/dark	2256	101	[62]
Me-4PACz	PSC	PCE 23.29 %	unencapsulated/nitrogen/dark/85 ℃	1008	60	[62]
Me-4PACz	PSC	PCE 23.29 %	unencapsulated/air/40~60 % RH	600	91	[62]
I-2PACz	PSC	PCE 25.39 %	unencapsulated/nitrogen/45 ℃	1000	96	[63]
MeO-2PACz/Me-4PACz	PSC	PCE 20.63 %	unencapsulated/nitrogen/65 ℃	2100	80.84	[64]
PANDI	PSC	PCE 21.5 %	unencapsulated/nitrogen/dark/27 ℃	1000	84	[65]
PANDI	PSC	PCE 21.5 %	unencapsulated/nitrogen/dark/65 ℃	1000	72	[65]
EA	PSC	PCE 24.17 %	MPP/encapsulated/nitrogen/30 ℃	460	90	[66]
MeO-2PACz	Tin-PSC	PCE 9.4 %	-	-	-	[67]

2PACz/KF	PSC	PCE 23.25 %	unencapsulated/nitrogen/25 ℃	1400	90	[68]
2PACz/KF	PSC	PCE 23.25 %	unencapsulated/air/25 ℃/50~75 % RH	1400	80	[68]
HBzA/2PACz	PSC	PCE 25.6 %	encapsulated/air/1 sun/85 ℃/50 % RH	1000	95	[69]
HBzA/2PACz	PSC	PCE 25.6 %	MPP/encapsulated/1 sun/40 ℃	1000	90	[69]
TDPA-Cl	PSC	PCE 22.4 %	-	-	-	[70]
Cbz2SMe	PSC	PCE 24.42 %	unencapsulated/nitrogen	1536	93	[71]
Cbz2SMe	PSC	PCE 24.42 %	unencapsulated/nitrogen/74 ± 4 % RH	910	92	[71]
2PACz	PeLED	EQE 4.42 % (467 nm)	15 cd m^{-2}	0.022	50	[72]
PPA	PSC	PCE 22.2 %	unencapsulated/air/35 ± 5 % RH	720	90	[73]
PPA	PSC	PCE 22.2 %	unencapsulated/nitrogen/60 ℃	960	60	[73]
9CAA	Tin-Lead PSC	PCE 23.1 %	nitrogen	2500	93	[74]
CbzBT	PSC	PCE 24 %	-	-	-	[75]
PC/Me-4PACz	PSC	PCE 25.09 %	encapsulated/air/55 ℃	1000	93	[76]
MeO-BTBT	PSC	PCE 24.53 %	MPP/encapsulated/nitrogen/40 ℃	1000	94.2	[77]
Ph-4PACz	PSC	PCE 25.6 %	MPP/unencapsulated/nitrogen/65 ± 5 ℃	1000	91.7	[78]
2BrPXZPA	PSC	PCE 22.93 %	MPP	600	97	[79]
Z907	PSC	PCE 20.4 %	MPP	550	46	[80]
Z907	PSC	PCE 20.4 %	unencapsulated/10 % RH	1000	80	[80]
Z907	PSC	PCE 20.4 %	encapsulated/20~25 ℃/32 % RH	1000	80	[80]
Z907	PSC	PCE 20.4 %	encapsulated/20~25 ℃/60 % RH	600	60	[80]
Z907	PSC	PCE 20.4 %	encapsulated/nitrogen/60 ℃	1200	60	[80]
CPTPB/PCBA	PSC	PCE 24.8 %	MPP/nitrogen	700	85	[81]
CPTPB/PCBA	PSC	PCE 24.8 %	unencapsulated/25 ℃/20~30 % RH	5000	90	[81]
CPTPB/PCBA	PSC	PCE 24.8 %	unencapsulated/nitrogen/85 ℃	1000	95	[81]
CPTPB/PCBA	PSC	PCE 24.8 %	unencapsulated/nitrogen/85 ℃/85 % RH	100	90	[81]
AB	Tin-PSC	PCE 0.076 %	unencapsulated/ ~60 % RH/dark	3500	80	[82]

TQxD	Tin-PSC	PCE 8.3 %	nitrogen/dark	1600	90	[83]
TQx	Tin-PSC	PCE 8.0 %	-	-	-	[83]
PQxD	Tin-PSC	PCE 7.1 %	nitrogen/dark	1600	90	[83]
PQx	Tin-PSC	PCE 6.1 %	-	-	-	[83]
MeO-2PACz	Tin-PSC	PCE 5.8 %	nitrogen/dark	336	-	[84]
MeO-2PACz	PSC (single-crystal)	PCE 23.1 %	MPP/unencapsulated/nitrogen	730	75	[85]
2PACz	Si/P TSC	PCE 32.5 %	MPP/encapsulated/air/50~60 % RH	870	90	[86]
4dp3PACz	PTSC	PCE 26.5 %	MPP/encapsulated/nitrogen	3000	70	[87]
DBzFe	PSC	PCE 23.53 %	MPP/unencapsulated/nitrogen	400	94.6	[88]
CZPC	PeLED	EQE 5.42 %（488 nm）	100 cd m^{-2}	6.1	50	[89]
L-Asp	PSC	PCE 18.46 %	-	-	-	[90]
MeO-2PACz	Tin-PSC	PCE 12.16 %	unencapsulate/air/25 °C/30~50 % RH	300	92.6	[91]
2PACz	PSC	PCE 24.5 %	MPP/encapsulate/air/~60 % RH	400	95	[92]
PPA	PSC	PCE 23.24 %	MPP/ unencapsulate/nitrogen/ 55 ± 5 °C	1000	91	[93]
MeO-2PACz	PSC	PCE 21.7 %	air/25 °C/20~30 % RH	800	82	[94]
4PADCB	PTSC	PCE 27 % (1.044 cm^2)	MPP/encapsulated	415	80	[95]
Pz:PFN	PSC	PCE 20.14 %	unencapsulation/nitrogen/40~50 % RH	3000	95	[96]
PHTCA	Quasi-2D PSC	PCE 17.74 %	MPP/encapsulated/air/60 % RH	1000	80	[97]
AC-5	PSC	PCE 23.19 %	MPP/nitrogen	600	88	[98]
Me-4PACz	PSC	PCE 19.5 %	unencapsulated/nitrogen	3600	93	[99]
2PADBC	Tin-PSC	PCE 14.19 %	MPP/encapsulated/nitrogen	1000	93	[100]
MeO-PhPACz	PSC	PCE 21.10 %	MPP/ unencapsulated/nitrogen	1000	85	[101]
Spiro-Acid	PSC	PCE 18.15 %	MPP/nitrogen	100	-	[102]
2PACz	PeLED	EQE 4.87 % (512 nm)	-	-	-	[103]
2PACz	PeLED	EQE14.5 % (493 nm)	-	-	-	[104]

2PACz	PeLED	EQE14.5 % (515 nm)	-	-	-	[104]
MeO-4PADBC	PSC	PCE 25.6 %	MPP/encapsulated/25 °C	1200	85	[105]
amine-2PACz	PSC	PCE 21.6 - 22.0 %	-	-	-	[106]
CbzNaph	PSC	PCE 24.98 %	MPP/unencapsulated/nitrogen	700	82	[107]
LS2/PTAA-0.2P	PSC	PCE 24.5 % (19.4 cm^2)	MPP/encapsulated/air	1200	80	[108]
2PACz/MeO-2PACz	PSC	PCE 20.11 %	unencapsulated	1752	85	[109]
2PAC/3-MPA	PSC	PCE 24 %	MPP/encapsulated/65 °C/50 % RH	1000	95	[110]
Br-2PACz/4CzNH3 I	Tin-Lead PSC	PCE 19.45 %	MPP/encapsulated/air	358	80	[111]
Br-2PACz	PSC	PCE 19.51 %	nitrogen/dark	4224	80.2	[112]
Isonicotinic Acid	PSC	PCE 18.68 %	50 °C/1 sun	68	99	[113]
MeO-2PACz/C10-BTBT	PSC	PCE 20.5 %	MPP/air/45 ± 5 °C/50 ± 10 % RH	803	80	[114]
2PACz/MeO-2PACz	PSC	PCE 21.8 %	unencapsulated/nitrogen/85 ℃	1200	86	[115]
2PACz/DMSO	PTSC	PCE 24.66 %	MPP/unencapsulated/air/~30 % RH	100	90	[116]
Ph-2PACz	Si/P TSC	PCE 21.3 %	200 cycles between 40 °C and 85 °C	200	98.8	[117]
BCBBr-C4PA	PSC	PCE 18.63 %	MPP/unencapsulated/nitrogen	250	90	[118]
BCB-C4PA	PSC	PCE 22.2 %	unencapsulated/nitrogen	2750	90	[119]
MC-45	PSC	PCE 16.69 %	-	30	80	[120]
MC-54	PSC	PCE 19.52 %	-	30	80	[120]
MC-55	PSC	PCE 18.99 %	-	30	80	[120]
DCB-BPA	PSC	PCE 18.88 %	MPP/encapsulated/50 % RH	427	80	[121]
Me-4PACz	PSC	PCE 24.5 %	MPP/unencapsulated/nitrogen/40~50 °C	1200	91	[122]
Me-4PACz	PeLED	EQE 16.7 %（538 nm）	200 cd m^{-2}	0.58	50	[123]
CbzNaph	PSC	PCE 24.1 %	unencapsulated/nitrogen/1 sun	120	97	[124]
CbzNaph	PSC	PCE 24.1 %	unencapsulated/85 ℃	168	70	[124]
FPAC60/bis-DMEC60	PSC	PCE 22.58 % (1 cm^2)	encapsulated/60 ℃/30 % RH	1000	90	[125]
FPAC60/bis-DMEC60	PSC	PCE 22.58 % (1 cm^2)	MPP/encapsulated	1300	90	[125]

2PACz	PSC	PCE 22.2 %	MPP/unencapsulated/air/45 °C/40 % RH	400	90	[126]
2PACz	PSC	PCE 22.2 %	MPP/encapsulated/air/45 °C/40 % RH	2000	82	[126]
Br-2EPSe	PSC	PCE 22.73 %	MPP/unencapsulated/air/30 °C/15~25 % RH	500	96	[127]
MPA-Ph-CA	PSC	PCE 22.53 %	dark/20~30 % RH	1500	100	[128]
MPA-Ph-CA	PSC	PCE 22.53 %	MPP/nitrogen/45 °C	800	95	[128]
36ClCzEPA	PeLED	EQE 4.80 % (473 nm)	100 cd m^{-2}	0.041	50	[129]
Br-2PACz	PeLED	EQE 18.57 % (515 nm)	100 cd m^{-2}	0.308	50	[130]
TBAC	PSC	PCE 23.50 %	unencapsulated/air/dark/25 °C/10 % RH	1000	90	[131]
2-TA	PSC	PCE 20.6 %	unencapsulated/20 % RH	480	80	[132]
2PACz/MeO-2PACz	Flexible P/P TSC	PCE 24.7 %	unencapsulated/nitrogen/dark	1000	100	[133]
2PACz/MeO-2PACz	Flexible P/P TSC	PCE 24.7 %	MPP/encapsulated/40~70 % RH	150	90	[133]
IAHA/DC-PA	PSC	PCE 23.59 %	nitrogen/65 °C	450	97	[134]
MPA-BT-CA	PSC	PCE 21.81 %	unencapsulated/45 % RH	480	60	[135]
MPA-BT-CA	PSC	PCE 21.81 %	unencapsulated/85 °C	144	60	[135]
RC-24	PSC	PCE 19.8 %	-	-	-	[136]
RC-25	PSC	PCE 19.6 %	-	-	-	[136]
RC-34	PSC	PCE 19.7 %	-	-	-	[136]
CBSA	PSC	PCE 21.8 %	unencapsulated/nitrogen/85 °C	800	80	[137]
CBSA	PSC	PCE 21.8 %	unencapsulated/nitrogen/25 °C	3000	85	[137]
CBSA	PSC	PCE 21.8 %	unencapsulated/air/50~70 % RH	1000	81	[137]
MPA/2PACz	Tin-Lead PSC	PCE 23.3 %	nitrogen/1 sun	1000	1000	[138]
2PACz	Si/P TSC	PCE 27.6 %	-	-	-	[139]
LS1	PSC	PCE 20.94 %	MPP/encapsulated	600	93	[140]
DPAH	PSC	PCE 21.44 %	unencapsulated/nitrogen/60 °C	1272	90	[141]
CDSC	PSC	PCE 22.22 %	unencapsulated/nitrogen/60 °C	1272	90	[141]
DPAH	PSC	PCE 21.44 %	unencapsulated/dark/10~20 % RH	1416	96.7	[141]

CDSC	PSC	PCE 22.22 %	unencapsulated/dark/10~20 % RH	1416	98.2	[141]
Br-2EPT	PSC	PCE 22.44 %	MPP/unencapsulated/15~25 % RH	100	100	[142]
PFDT	PSC	PCE 21.79 %	MPP/unencapsulated/nitrogen/85 °C	500	90.1	[143]
PFDT	PSC	PCE 21.79 %	unencapsulated/air/85 ± 10 % RH	500	95	[143]
P3CT-Na	PSC	PCE 20.87 %	unencapsulated/nitrogen	1200	92	[144]
4MP	PeLED	EQE 5.74 % (532 nm)	-	4.545	50	[145]
4ATP	PeLED	EQE 4.75 % (532 nm)	-	3.758	50	[145]
CA	PeLED	EQE 4.21 % (532 nm)	-	2.343	50	[145]
TP	PeLED	EQE 2.63 % (532 nm)	-	1.848	50	[145]
EADR03	PSC	PCE 21.2 %	MPP/unencapsulated/nitrogen/25 °C	183	80	[146]
EADR04	PSC	PCE 21.0 %	MPP/unencapsulated/nitrogen/25 °C	872	80	[146]
EADR04	PSC	PCE 21.0 %	MPP/unencapsulated/nitrogen/85 °C	242	80	[146]
4-CLBA	PSC	PCE 21 %	unencapsulated/air/25 °C/50 ± 5 % RH	700	80	[147]
TSPA	PSC	PCE 20.21 %	unencapsulated/25 °C/30~40 % RH	600	95	[148]
TSPA	PSC	PCE 20.21 %	unencapsulated/25 °C/50~60 % RH	1440	75	[148]
I-SAM	PSC	PCE 21.4 %	MPP/unencapsulated/nitrogen	3006/1896/3921	80	[149]
MPTMS	PSC	PCE 20.03 %	unencapsulated/air/15 °C/30 % RH	720	90	[150]
P3HT-COOH	PSC	PCE 19.21 %	unencapsulated/30 % RH	4300	80	[151]
P3HT-COOH	PSC	PCE 19.21 %	unencapsulated/55 % RH	700	80	[151]
P3HT-COOH	PSC	PCE 19.21 %	unencapsulated/nitrogen/65 °C	470	80	[151]
P3HT-COOH	PSC	PCE 19.21 %	unencapsulated/nitrogen/1 sun	220	80	[151]
Me-4PACz	Si/P TSC	PCE 29.15 %	MPP/unencapsulated/air/25 °C/30~40 % RH	300	95.5	[152]
Creatine	PSC	PCE 22.1 %	unencapsulated/30~40 % RH	1200	90	[153]
PTABr	PSC	PCE 20.13 %	unencapsulated/60~80 % RH	1440	86	[154]
ADAI	PSC	PCE 15.9 %	-	-	-	[155]
EPA	PSC	PCE 13.0 %	unencapsulated/75 °C	7.1	80	[156]

BBA	PSC	PCE 12.6 %	unencapsulated/75 ℃	8.3	80	[156]
CPTA	PSC	PCE 18.12 %	MPP/encapsulated/25 ℃	1000	100	[157]
CPTA	PSC	PCE 18.12 %	MPP/encapsulated/60 ℃	1000	80	[157]
choline chloride	PSC	PCE 18.9 %	-	-	-	[158]
I-PFC10	PSC	PCE 21.3 %	unencapsulated/air/30~50 % RH	3000	95	[159]
I-PFC10	PSC	PCE 21.3 %	MPP/unencapsulated/nitrogen/85 ℃	250	75	[159]
TMBA	PSC	PCE 13.75 %	unencapsulated/dark/28 ℃/45 % RH	168	66.81	[160]
ATAA	PSC	PCE 19.74 %	-	-	-	[161]
CBA	PSC	PCE 21.35 %	MPP/unencapsulated	1440	90	[162]
MBA	PSC	PCE 20.73 %	MPP/unencapsulated	1440	80	[162]
β-ALA	PSC	PCE 19.43 %	-	-	-	[162]
NBA	PSC	PCE 1.11 %	-	-	-	[162]
NDI-P	PSC	PCE 16 %	unencapsulated/1 sun	500	82	[163]
2PACz	PSC	PCE 20.8 %	MPP/40 ℃	11	97	[164]
MeO-2PACz	PSC	PCE 20.2 %	MPP/40 ℃	11	97	[164]
V1036	PSC	PCE 16.9 %	MPP/40 ℃	11	88	[164]
2D-PT	PSC	PCE 14.8 %	unencapsulated/25 ℃/90 % RH	24	85	[165]
P3HT	PSC	PCE 11.3 %	unencapsulated/25 ℃/90 % RH	24	68.9	[165]
C3-SAM	PSC	PCE 14.7 %	-	-	-	[166]
4-PA	PSC	PCE 18.9 %	-	-	-	[167]
MC-43	PSC	PCE 17.3 %	encapsulated/dark	480	90	[168]
3-PA	PSC	PCE 16.88 %	-	-	-	[169]
V1036	PSC	PCE 17.8 %	nitrogen/dark	4320	94	[170]
C60-SAM	PSC	PCE 17.8 %	unencapsulated/nitrogen/30 % RH	180	95	[171]
DA	PSC	PCE 16.65 %	air/70 % RH	288	80	[172]
2F-SAM	PSC	PCE 16 %	unencapsulated	720	80	[173]

T2CA	PSC	PCE 17.07 %	-	-	-	[174]
JTCA	PSC	PCE 18.82 %	unencapsulated/air/1 sun	840	70	[174]
C6-PA	PSC	PCE 16.6 %	-	-	-	[175]

Reference

1. He J, Li G, Huang G *et al.* Improved Anchoring of Self-Assembled Monolayer on Hydroxylated NiOx Film Surface for Efficient and Stable Inverted Perovskite Solar Cells. *Adv Funct Mater* 2024:2413104.

2. Chen C-H, Liu G-W, Chen X *et al.* Methylthio Substituent in SAM Constructing Regulatory Bridge with Photovoltaic Perovskites. *Angew Chem Int Ed* 2024:e202419375.

3. Wu T, Raju TB, Shang J *et al.* Lattice Matching Anchoring of Hole-Selective Molecule on Halide Perovskite Surfaces for n-i-p Solar Cells. *Adv Mater* 2024:2414576.

4. Lee HJ, Do JJ, Jung JW. Enhanced Hole-Injecting Interface for High-Performance Deep-Blue Perovskite Light-Emitting Diodes Using Dipole-Controlled Self-Assembled Monolayers. *Small* 2024:2407769.

5. Chen B, Peng C, Guo R *et al.* Dual-Function Self-Assembled Molecules as Hole-Transport Layers for Thermally Evaporated High-Efficiency Blue Perovskite Light-Emitting Diodes. *Adv Mater* 2024:2411451.

6. Zhang Q, Wang H, Zhao Q *et al.* Machine-Learning-Assisted Design of Buried-Interface Engineering Materials for High-Efficiency and Stable Perovskite Solar Cells. *ACS Energy Lett* 2024:5924–34.

7. Afraj SN, Kuan C-H, Cheng H-L *et al.* Triphenylamine-Based Y-Shaped Self-Assembled Monolayers for Efficient Tin Perovskite Solar Cells. *Small* 2024:2408638.

8. Huang Y, Tao M, Zhang Y *et al.* Asymmetric Modification of Carbazole Based Self-Assembled Monolayers by Hybrid Strategy for Inverted Perovskite Solar Cells. *Angew Chem Int Ed* 2024:e202416188.

9. Su Z, Cui M, Dong B *et al.* Stereo-Hindrance Induced Conformal Self-Assembled Monolayer for High Efficiency Inverted Perovskite Solar Cells. *Small* 2024:2407387.

10. Lan Z-R, Ma D-X, Tang K *et al.* Indolo[3,2-b]carbazole-Based Self-Assembled Monolayer Enables High-Performance Inverted Perovskite Solar Cells with Over 25.5% Efficiency. *CCS Chem* 2024;**0**:1–9.

11. Jiang W, Wang D, Shang W *et al.* Spin-Coated and Vacuum-Processed Hole-Extracting Self-Assembled Multilayers with H-Aggregation for High-Performance Inverted Perovskite Solar Cells. *Angew Chem Int Ed* 2024;**63**:e202411730.

12. Harter A, Artuk K, Mathies F *et al.* Perovskite/Silicon Tandem Solar Cells Above 30% Conversion Efficiency on Submicron-Sized Textured Czochralski-Silicon Bottom Cells with Improved Hole-Transport Layers. *ACS Appl Mater Interfaces* 2024;**16**:62817–26.

13. Zhang Y, Liu Y, Zhao Z *et al.* Improving Buried Interface Contact for Inverted Perovskite Solar Cells via Dual Modification Strategy. *Adv Funct Mater* 2024:2417575.

14. Tang Y, Zhang Z, Liu H *et al.* Multifunctional Action Site Strategy of a Buried Interface for High-Performance Perovskite Solar Cells. *ACS Photonics* 2024;**11**:4916–22.

15. Yu M-H, Liu X, Yu H-W *et al.* Impact of self-assembled monolayer structural design on perovskite phase regulation, hole-selective contact, and energy loss in inverted perovskite solar cells. *Nano Energy* 2024;**132**:110405.

16. Lenaers S, Lammar S, Krishna A *et al.* Pyrene-Based Self-Assembled Monolayer with Improved Surface Coverage and Energy Level Alignment for Perovskite Solar Cells. *Adv Funct Mater* 2024:2411922.

17. Zhou L, Yan M, Luo G *et al.* Bottom Electrode Modification Enables Efficient and Bright Silicon-Based Top-Emission Perovskite Light-Emitting Diodes. *Small* 2024:2404181.

18. Zhu W, Yang M, Han T *et al.* Homogeneous crystallization of MA-free, wide-bandgap perovskite films via self-assembled monolayer capping for laminated silicon/perovskite tandem solar cells. *Chem Eng J* 2024;**500**:156798.

19. Chen X, Chen C-H, Su ZH *et al.* Adhesively Bridging SAM Molecules and Perovskites for Highly Efficient Photovoltaics. *Adv Funct Mater* 2024:2415004.

20. Tang Y, Dong X, Li X *et al.* Two-dimensional Ruddlesden-Popper perovskite solar cells with an efficiency exceeding 19 % by inserting a self-assembled monolayer. *Chem Eng J* 2024;**499**:156503.

21. Zhao K, Yao L, Liu C *et al.* Tailoring the π-conjugation in self-assembled hole-selective molecules for perovskite photovoltaics. *Sci China Mater* 2024, DOI: 10.1007/s40843-024-3093-9.

22. Pininti AR, Subbiah AS, Deger C *et al.* Resolving Scaling Issues in Self-Assembled Monolayer-Based Perovskite Solar Modules via Additive Engineering. *Adv Energy Mater* 2024:2403530.

23. Zhang C, Liang C, Sun F *et al.* Buried interface bridging for inverted cesium-formamidinium triiodide perovskite solar cells with long operational stability. *Sci China Chem* 2024, DOI: 10.1007/s11426-024-2234-x.

24. Xu Z, Wang J, Liu Z *et al.* Self-Assembled Monolayer Suppresses Interfacial Reaction between NiOx and Perovskite for Efficient and Stable Inverted Inorganic Perovskite Solar Cells. *ACS Appl Mater Interfaces* 2024;**16**:53811–21.

25. Soliman AIA, Zhang Y, Zhang L *et al.* Surface Reconstruction of Perovskites with Organosilanes for High Performance and Highly Stable Solar Cells. *Adv Funct Mater* 2024:2412886.

26. Wang X, Li J, Guo R *et al.* Regulating phase homogeneity by self-assembled molecules for enhanced efficiency and stability of inverted perovskite solar cells. *Nat Photonics* 2024;**18**:1269–75.

27. Dou Y, Lv P, Yuan Z *et al.* Enhanced Buried Interface Engineering for Efficient Inverted Perovskite Solar Cells Fabricated via Vapor–Solid Reaction. *Small Methods* 2024:2401339.

28. Kim TH, Kim BW, Im SH. A Self-Assembling Molecule for Improving the Mobility in PEDOT:PSS Hole Transport Layer for Efficient Perovskite Light-Emitting Diodes. *Adv Electron Mater* 2024:2400626.

29. Zou G, Zhu Z, Zeng Z *et al.* Self-Organized Carbazole Phosphonic Acid Additives at Buried Interface Enhance Efficiency of Blue Perovskite LEDs. *ACS Energy*

Lett 2024;**9**:4715–23.

30. Li D, Lian Q, Du T *et al.* Co-adsorbed self-assembled monolayer enables high-performance perovskite and organic solar cells. *Nat Commun* 2024;**15**:7605.

31. Kuan C-H, Afraj SN, Huang Y-L *et al.* Functionalized Thienopyrazines on NiOx Film as Self-Assembled Monolayer for Efficient Tin-Perovskite Solar Cells Using a Two-Step Method. *Angew Chem Int Ed* 2024;**63**:e202407228.

32. Li N, Xia Y, Lou Y-H *et al.* Dual-Functional Self-Assembled Molecule Enabling High-Performance Deep-Blue Perovskite Light-Emitting Diodes. *Adv Funct Mater* 2024:2411227.

33. Guo S, Yang X, Zhang Q *et al.* Fused Carbazole-based Self-Assembled Monolayers Enable Efficient Perovskite Solar Cells and Perovskite Light-Emitting Diodes. *Adv Opt Mater* 2024;**12**:2401617.

34. Roe J, Son JG, Park S *et al.* Synergistic Buried Interface Regulation of Tin–Lead Perovskite Solar Cells via Co-Self-Assembled Monolayers. *ACS Nano* 2024;**18**:24306–16.

35. Wan J, Zhang Z, Lin J *et al.* Simultaneously enhancing hole extraction and defect passivation with more conductive hole-selective self-assembled molecules for efficient inverted perovskite solar cells. *J Mater Chem C* 2024;**12**:15644–53.

36. Dong L, Qiu S, Feroze S *et al.* Simplifying contact-layer design for high-throughput printing of flexible perovskite photovoltaics. *Energy Environ Sci* 2024;**17**:7147–54.

37. Guo Y, Guo S, Wu T *et al.* Ultrafast hole transfer mediated by a conjugated self-assembled molecule enables efficient and stable wide-bandgap perovskite solar cells. *Chem Eng J* 2024;**497**:154722.

38. Ji H, Ren Z, Chen R *et al.* Tailoring Nio/Perovskite Interface via a Multifunctional Self-Assembled Molecule for High-Performance Blue Perovskite Light-Emitting Diodes. *Small Struct* 2024;**5**:2400153.

39. Hung C-M, Wu C-C, Yang Y-H *et al.* Repairing Interfacial Defects in Self-Assembled Monolayers for High-Efficiency Perovskite Solar Cells and Organic

Photovoltaics through the SAM@Pseudo-Planar Monolayer Strategy. *Adv Sci* 2024;**11**:2404725.

40. Tong X, Xie L, Li J *et al.* Large Orientation Angle Buried Substrate Enables Efficient Flexible Perovskite Solar Cells and Modules. *Adv Mater* 2024;**36**:2407032.

41. Qu G, Cai S, Qiao Y *et al.* Conjugated linker-boosted self-assembled monolayer molecule for inverted perovskite solar cells. *Joule* 2024;**8**:2123–34.

42. Li J, Xie L, Yang S *et al.* Self-assembling Monolayer-Assisted Perovskite Growth Enables High-Performance Solar Cells. *Chin J Chem* 2024;**42**:2795–803.

43. Li T, Wang K, Tong Y *et al.* In Situ Dehydration Condensation of Self-Assembled Molecules Enables Stabilization of CsPbI3 Perovskites for Efficient Photovoltaics. *Adv Funct Mater* 2024:2409621.

44. Zhou H, Wang W, Duan Y *et al.* Glycol Monomethyl Ether-Substituted Carbazolyl Hole-Transporting Material for Stable Inverted Perovskite Solar Cells with Efficiency of 25.52 %. *Angew Chem Int Ed* 2024;**63**:e202403068.

45. Liu S, Li J, Xiao W *et al.* Buried interface molecular hybrid for inverted perovskite solar cells. *Nature* 2024;**632**:536–42.

46. Wang J, Liu N, Liu Z *et al.* Versatile Self-Assembled Monolayer Enables High-Performance Inverted CsPbI3 Perovskite Solar Cells. *ACS Appl Nano Mater* 2024;**7**:15267–76.

47. Li C, Li Y, Chen Y *et al.* Enhancing Efficiency of Industrially-Compatible Monolithic Perovskite/Silicon Tandem Solar Cells with Dually-Mixed Self-Assembled Monolayers. *Adv Funct Mater* 2024;**34**:2407805.

48. Wang Y, Ye J, Song J *et al.* Synchronous modulation of hole-selective self-assembled monolayer and buried interface for inverted perovskite solar cells. *Cell Rep Phys Sci* 2024;**5**, DOI: 10.1016/j.xcrp.2024.101992.

49. Zhu H, Xu Z, Zhang Z *et al.* Improved Hole-Selective Contact Enables Highly Efficient and Stable FAPbBr3 Perovskite Solar Cells and Semitransparent Modules. *Adv Mater* 2024;**36**:2406872.

50. Zhao Y, Luan X, Han L *et al.* Post-Assembled Alkylphosphonic Acids for Efficient and Stable Inverted Perovskite Solar Cells. *Adv Funct Mater*

2024;**34**:2405646.

51. Cao K, Ning H, Xu N *et al.* Tailoring the buried interface with self-assembled 2-chloroethylphosphonic acid for defect reduction and improved performance of tin-based perovskite solar cells. *J Mater Chem A* 2024;**12**:17444–52.

52. Guo W, Li J, Cen H *et al.* Self-Assembled Molecules Fostering Ordered Spatial Heterogeneity for Efficient Ruddlesden-Popper Perovskite Solar Cells. *Adv Energy Mater* 2024;**14**:2401303.

53. Dong L, Lv P, Zhu B *et al.* Self-assembled complexing agent assisted chemical bath deposition of SnOX enabled highly efficient perovskite solar cells. *Chem Eng J* 2024;**493**:152378.

54. Wu J, Yan P, Yang D *et al.* Bisphosphonate-Anchored Self-Assembled Molecules with Larger Dipole Moments for Efficient Inverted Perovskite Solar Cells with Excellent Stability. *Adv Mater* 2024;**36**:2401537.

55. Yuan X, Ling X, Wang H *et al.* Modulation on electrostatic potential to build a firm bridge at NiO_x/perovskite interface for efficient and stable perovskite solar cells. *J Energy Chem* 2024;**96**:249–58.

56. Zhang C, Son Y, Kim H *et al.* Work function tuning of a weak adhesion homojunction for stable perovskite solar cells. *Joule* 2024;**8**:1394–411.

57. Sun X, Fan H, Xu X *et al.* A Fluorination Strategy and Low-Acidity Anchoring Group in Self-Assembled Molecules for Efficient and Stable Inverted Perovskite Solar Cells. *Chem – Eur J* 2024;**30**:e202400629.

58. Chen J, Zhang X, Liu X *et al.* A Multifunctional Dye Molecule as the Interfacial Layer for Perovskite Solar Cells. *ACS Appl Mater Interfaces* 2024;**16**:22079–88.

59. Zhou Y, Huang X, Zhang J *et al.* Interfacial Modification of NiOx for Highly Efficient and Stable Inverted Perovskite Solar Cells. *Adv Energy Mater* 2024;**14**:2400616.

60. Sukhorukova PK, Ilicheva EA, Gostishchev PA *et al.* Triphenylamine-based interlayer with carboxyl anchoring group for tuning of charge collection interface in stabilized p-i-n perovskite solar cells and modules. *J Power Sources* 2024;**604**:234436.

61. Gao M, Xu X, Tian H *et al.* Enhancing Efficiency of Large-Area Wide-Bandgap Perovskite Solar Modules with Spontaneously Formed Self-Assembled Monolayer Interfaces. *J Phys Chem Lett* 2024;**15**:4015–23.

62. Yang B, Cai B, Zhou T *et al.* Facile and sustainable interface modulation *via* a self-assembly phosphonate molecule for efficient and stable perovskite photovoltaics. *Chem Eng J* 2024;**488**:150861.

63. Dai Z, You S, Chakraborty D *et al.* Connecting Interfacial Mechanical Adhesion, Efficiency, and Operational Stability in High Performance Inverted Perovskite Solar Cells. *ACS Energy Lett* 2024;**9**:1880–7.

64. Kim D-H, Lee H-J, Lee S-H *et al.* Mixed Self-Assembled Hole-Transport Monolayer Enables Simultaneous Improvement of Efficiency and Stability of Perovskite Solar Cells. *Sol RRL* 2024;**8**:2400067.

65. Utomo DS, Svirskaite LM, Prasetio A *et al.* Nonfullerene Self-Assembled Monolayers As Electron-Selective Contacts for n-i-p Perovskite Solar Cells. *ACS Energy Lett* 2024;**9**:1682–92.

66. Zhang Y, Kong T, Liu Y *et al.* The Effect of Self-Assembled Bridging Layer on the Performance of Pure FAPbI3-Based Perovskite Solar Cells. *Adv Funct Mater* 2024;**34**:2401391.

67. Song D, Ramakrishnan S, Zhang Y *et al.* Mixed Self-Assembled Monolayers for High-Photovoltage Tin Perovskite Solar Cells. *ACS Energy Lett* 2024;**9**:1466–72.

68. Wang S, Khan D, Zhou W *et al.* Ion-Dipole Interaction for Self-Assembled Monolayers: A New Strategy for Buried Interface in Inverted Perovskite Solar Cells. *Adv Funct Mater* 2024;**34**:2316202.

69. Azmi R, Utomo DS, Vishal B *et al.* Double-side 2D/3D heterojunctions for inverted perovskite solar cells. *Nature* 2024;**628**:93–8.

70. Li M, Li Z, Liu M *et al.* A Hole-Selective Self-Assembled Monolayer for Both Efficient Perovskite and Organic Solar Cells. *Langmuir* 2024;**40**:4772–8.

71. Jiang W, Hu Y, Li F *et al.* Hole-Selective Contact with Molecularly Tailorable Reactivity for Passivating High-Performing Inverted Perovskite Solar Cells. *CCS*

Chem 2024;**6**:1654–61.

72. Zheng X, Wei C, Yuan S *et al.* Self-assembled mono-hole-injecting layers towards efficient deep-blue perovskite light-emitting diodes. *Appl Surf Sci* 2024;**655**:159643.

73. Wu Y, Wang Y, Song J *et al.* Multifunctional molecular linker on buried interface for efficient and stable cesium–formamidinium perovskite solar cells. *Appl Phys Lett* 2024;**124**:073907.

74. Zhang Z, Zhu R, Tang Y *et al.* Anchoring Charge Selective Self-Assembled Monolayers for Tin–Lead Perovskite Solar Cells. *Adv Mater* 2024;**36**:2312264.

75. Jiang W, Liu M, Li Y *et al.* Rational molecular design of multifunctional self-assembled monolayers for efficient hole selection and buried interface passivation in inverted perovskite solar cells. *Chem Sci* 2024;**15**:2778–85.

76. Cao Q, Wang T, Pu X *et al.* Co-Self-Assembled Monolayers Modified NiO for Stable Inverted Perovskite Solar Cells. *Adv Mater* 2024;**36**:2311970.

77. Liu M, Li M, Li Y *et al.* Defect-Passivating and Stable Benzothiophene-Based Self-Assembled Monolayer for High-Performance Inverted Perovskite Solar Cells. *Adv Energy Mater* 2024;**14**:2303742.

78. Sun A, Tian C, Zhuang R *et al.* High Open-Circuit Voltage (1.197 V) in Large-Area (1 cm2) Inverted Perovskite Solar Cell via Interface Planarization and Highly Polar Self-Assembled Monolayer. *Adv Energy Mater* 2024;**14**:2303941.

79. Li Z, Tan Q, Chen G *et al.* Simple and robust phenoxazine phosphonic acid molecules as self-assembled hole selective contacts for high-performance inverted perovskite solar cells. *Nanoscale* 2023;**15**:1676–86.

80. Liu L, Yang Y, Du M *et al.* Self-Assembled Amphiphilic Monolayer for Efficient and Stable Wide-Bandgap Perovskite Solar Cells. *Adv Energy Mater* 2023;**13**:2202802.

81. Chen Z, Li Y, Liu Z *et al.* Reconfiguration toward Self-Assembled Monolayer Passivation for High-Performance Perovskite Solar Cells. *Adv Energy Mater* 2023;**13**:2202799.

82. Abid A, Rajamanickam P, Wei-Guang Diau E. Design of a simple bifunctional system as a self-assembled monolayer (SAM) for inverted tin-based perovskite solar cells. *Chem Eng J* 2023;**477**:146755.

83. Afraj SN, Kuan C-H, Lin J-S *et al.* Quinoxaline-Based X-Shaped Sensitizers as Self-Assembled Monolayer for Tin Perovskite Solar cells. *Adv Funct Mater* 2023;**33**:2213939.

84. Aktas E, Poli I, Ponti C *et al.* One-Step Solution Deposition of Tin-Perovskite onto a Self-Assembled Monolayer with a DMSO-Free Solvent System. *ACS Energy Lett* 2023;**8**:5170–4.

85. Almasabi K, Zheng X, Turedi B *et al.* Hole-Transporting Self-Assembled Monolayer Enables Efficient Single-Crystal Perovskite Solar Cells with Enhanced Stability. *ACS Energy Lett* 2023;**8**:950–6.

86. Aydin E, Ugur E, Yildirim BK *et al.* Enhanced optoelectronic coupling for perovskite/silicon tandem solar cells. *Nature* 2023;**623**:732–8.

87. Bi H, Liu J, Zhang Z *et al.* All-Perovskite Tandem Solar Cells Approach 26.5% Efficiency by Employing Wide Bandgap Lead Perovskite Solar Cells with New Monomolecular Hole Transport Layer. *ACS Energy Lett* 2023;**8**:3852–9.

88. Bi H, Liu J, Zhang Z *et al.* Ferrocene Derivatives for Improving the Efficiency and Stability of MA-Free Perovskite Solar Cells from the Perspective of Inhibiting Ion Migration and Releasing Film Stress. *Adv Sci* 2023;**10**:2304790.

89. Chen B, Guo R, He Z *et al.* Self-assembled monolayers as hole transport layers for efficient thermally evaporated blue perovskite light-emitting diodes. *Chem Eng J* 2023;**476**:146476.

90. Xiang C, Junjie C, Pengyun Z *et al.* Efficient perovskite solar cells by interface optimization with l-aspartic acid in air atmosphere. *Org Electron* 2023;**122**:106904.

91. Cho S, Pandey P, Yoon S *et al.* Anchoring self-assembled monolayer at perovskite/hole collector interface for wide bandgap Sn-based solar cells with a record efficiency over 12%. *Surf Interfaces* 2023;**42**:103478.

92. Guo R, Wang X, Jia X *et al.* Refining the Substrate Surface Morphology for Achieving Efficient Inverted Perovskite Solar Cells. *Adv Energy Mater* 2023;**13**:2302280.

93. Guo R, Zhang X, Zheng X *et al.* Tailoring Multifunctional Self-Assembled Hole Transporting Molecules for Highly Efficient and Stable Inverted Perovskite Solar Cells. *Adv Funct Mater* 2023;**33**:2211955.

94. Guo Y, Huang L, Wang C *et al.* Efficient inverted perovskite solar cells with a low-temperature processed NiO x /SAM hole transport layer. *J Mater Chem C* 2024;**12**:1507–15.

95. He R, Wang W, Yi Z *et al.* Improving interface quality for 1-cm2 all-perovskite tandem solar cells. *Nature* 2023;**618**:80–6.

96. Hossain K, Kulkarni A, Bothra U *et al.* Resolving the Hydrophobicity of the Me-4PACz Hole Transport Layer for Inverted Perovskite Solar Cells with Efficiency >20%. *ACS Energy Lett* 2023;**8**:3860–7.

97. Huang H-H, Yang T-A, Su L-Y *et al.* Thiophene-Based Polyelectrolyte Boosts High-Performance Quasi-2D Perovskite Solar Cells with Ultralow Energy Loss. *ACS Mater Lett* 2023;**5**:1384–94.

98. Hung C-M, Mai C-L, Wu C-C *et al.* Self-Assembled Monolayers of Bi-Functionalized Porphyrins: A Novel Class of Hole-Layer-Coordinating Perovskites and Indium Tin Oxide in Inverted Solar Cells. *Angew Chem Int Ed* 2023;**62**:e202309831.

99. Kulkarni A, Sarkar R, Akel S *et al.* A Universal Strategy of Perovskite Ink - Substrate Interaction to Overcome the Poor Wettability of a Self-Assembled Monolayer for Reproducible Perovskite Solar Cells. *Adv Funct Mater* 2023;**33**:2305812.

100. Li B, Zhang C, Gao D *et al.* Suppressing Oxidation at Perovskite–NiO Interface for Efficient and Stable Tin Perovskite Solar Cells. *Adv Mater* 2024;**36**:2309768.

101. Li C, Zhang Z, Zhang H *et al.* Fully Aromatic Self-Assembled Hole-Selective Layer toward Efficient Inverted Wide-Bandgap Perovskite Solar Cells with Ultraviolet Resistance. *Angew Chem Int Ed* 2024;**63**:e202315281.

102. Li W, Cariello M, Méndez M *et al.* Self-Assembled Molecules for Hole-Selective Electrodes in Highly Stable and Efficient Inverted Perovskite Solar Cells with Ultralow Energy Loss. *ACS Appl Energy Mater* 2023;**6**:1239–47.

103. Li W, Li T, Tong Y *et al.* Fabrication of Highly Luminescent Quasi Two-Dimensional CsPbBr3 Perovskite Films in High Humidity Air for Light-Emitting Diodes. *ACS Appl Mater Interfaces* 2023;**15**:36602–10.

104. Li Z, Chen Z, Shi Z *et al.* Charge injection engineering at organic/inorganic heterointerfaces for high-efficiency and fast-response perovskite light-emitting diodes. *Nat Commun* 2023;**14**:6441.

105. Li Z, Sun X, Zheng X *et al.* Stabilized hole-selective layer for high-performance inverted p-i-n perovskite solar cells. *Science* 2023;**382**:284–9.

106. Lin J, Wang Y, Khaleed A *et al.* Dual Surface Modifications of NiOx/Perovskite Interface for Enhancement of Device Stability. *ACS Appl Mater Interfaces* 2023;**15**:24437–47.

107. Liu M, Bi L, Jiang W *et al.* Compact Hole-Selective Self-Assembled Monolayers Enabled by Disassembling Micelles in Solution for Efficient Perovskite Solar Cells. *Adv Mater* 2023;**35**:2304415.

108. Niu B, Liu H, Huang Y *et al.* Multifunctional Hybrid Interfacial Layers for High-Performance Inverted Perovskite Solar Cells. *Adv Mater* 2023;**35**:2212258.

109. Ou Y, Huang H, Shi H *et al.* Collaborative interfacial modification and surficial passivation for high-efficiency MA-free wide-bandgap perovskite solar cells. *Chem Eng J* 2023;**469**:143860.

110. Park SM, Wei M, Lempesis N *et al.* Low-loss contacts on textured substrates for inverted perovskite solar cells. *Nature* 2023;**624**:289–94.

111. Pitaro M, Alonso JES, Di Mario L *et al.* Tuning the Surface Energy of Hole Transport Layers Based on Carbazole Self-Assembled Monolayers for Highly Efficient Sn/Pb Perovskite Solar Cells. *Adv Funct Mater* 2024;**34**:2306571.

112. Pitaro M, Sebastian Alonso J, Mario LD *et al.* A carbazole-based self-assembled monolayer as the hole transport layer for efficient and stable Cs 0.25 FA 0.75 Sn 0.5 Pb 0.5 I 3 solar cells. *J Mater Chem A* 2023;**11**:11755–66.

113. Sekimoto T, Yamamoto T, Takeno F *et al.* Perovskite Solar Cell Using Isonicotinic Acid as a Gap-Filling Self-Assembled Monolayer with High Photovoltaic Performance and Light Stability. *ACS Appl Mater Interfaces* 2023;**15**:33581–92.

114. Takhellambam D, Castriotta LA, Zanotti G *et al.* Enhancing Hole Transfer in Perovskite Solar Cell with Self-Assembled Monolayer by Introducing [1]Benzothieno [3,2-b][1]Benzothiophene Interlayer. *Sol RRL* 2023;**7**:2300658.

115. Tutundzic M, Zhang X, Lammar S *et al.* Toward Efficient and Fully Scalable Sputtered NiO-Based Inverted Perovskite Solar Modules via Co-Ordinated Modification Strategies. *Sol RRL* 2024;**8**:2300862.

116. Vidyasagar D, Yun Y, Yu Cho J *et al.* Surface-functionalized hole-selective monolayer for high efficiency single-junction wide-bandgap and monolithic tandem perovskite solar cells. *J Energy Chem* 2024;**88**:317–26.

117. Wang G, Zheng J, Duan W *et al.* Molecular engineering of hole-selective layer for high band gap perovskites for highly efficient and stable perovskite-silicon tandem solar cells. *Joule* 2023;**7**:2583–94.

118. Wang W, Liu X, Wang J *et al.* Versatile Self-Assembled Molecule Enables High-Efficiency Wide-Bandgap Perovskite Solar Cells and Organic Solar Cells. *Adv Energy Mater* 2023;**13**:2300694.

119. Wang W, Wei K, Yang L *et al.* Dynamic self-assembly of small molecules enables the spontaneous fabrication of hole conductors at perovskite/electrode interfaces for over 22% stable inverted perovskite solar cells. *Mater Horiz* 2023;**10**:2609–17.

120. Yalcin E, Aktas E, Mendéz M *et al.* Monodentate versus Bidentate Anchoring Groups in Self-Assembling Molecules (SAMs) for Robust p–i–n Perovskite Solar Cells. *ACS Appl Mater Interfaces* 2023;**15**:57153–64.

121. Yi Z, Wang W, He R *et al.* Achieving a high open-circuit voltage of 1.339 V in 1.77 eV wide-bandgap perovskite solar cells via self-assembled monolayers. *Energy Environ Sci* 2024;**17**:202–9.

122. Zheng X, Li Z, Zhang Y *et al.* Co-deposition of hole-selective contact and absorber for improving the processability of perovskite solar cells. *Nat Energy*

2023;**8**:462–72.

123. Zhou L, Yan M, Luo G *et al.* Self-Assembled Molecule Doping Enables High-Efficiency Hole-Transport-Layer-Free Perovskite Light-Emitting Diodes. *Adv Funct Mater* 2023;**33**:2303370.

124. Jiang W, Li F, Li M *et al.* π-Expanded Carbazoles as Hole-Selective Self-Assembled Monolayers for High-Performance Perovskite Solar Cells. *Angew Chem Int Ed* 2022;**61**:e202213560.

125. Zhang S, Li M, Zeng H *et al.* Grain Boundary and Buried Interface Suturing Enabled by Fullerene Derivatives for High-Performance Perovskite Solar Module. *ACS Energy Lett* 2022;**7**:3958–66.

126. Zhu X, Lau CFJ, Mo K *et al.* Inverted planar heterojunction perovskite solar cells with high ultraviolet stability. *Nano Energy* 2022;**103**:107849.

127. Ullah A, Park KH, Lee Y *et al.* Versatile Hole Selective Molecules Containing a Series of Heteroatoms as Self-Assembled Monolayers for Efficient p-i-n Perovskite and Organic Solar Cells. *Adv Funct Mater* 2022;**32**:2208793.

128. Zhang S, Wu R, Mu C *et al.* Conjugated Self-Assembled Monolayer as Stable Hole-Selective Contact for Inverted Perovskite Solar Cells. *ACS Mater Lett* 2022;**4**:1976–83.

129. Shin YS, Ameen S, Oleiki E *et al.* A Multifunctional Self-Assembled Monolayer for Highly Luminescent Pure-Blue Quasi-2D Perovskite Light-Emitting Diodes. *Adv Opt Mater* 2022;**10**:2201313.

130. Gedda M, Gkeka D, Nugraha MI *et al.* High-Efficiency Perovskite–Organic Blend Light-Emitting Diodes Featuring Self-Assembled Monolayers as Hole-Injecting Interlayers. *Adv Energy Mater* 2023;**13**:2201396.

131. Zhong H, Jia Z, Shen J *et al.* Surface treatment of the perovskite via self-assembled dipole layer enabling enhanced efficiency and stability for perovskite solar cells. *Appl Surf Sci* 2022;**602**:154365.

132. Wu Y, Song J, Wu X *et al.* Highly efficient and stable ZnO-based perovskite solar cells enabled by a self-assembled monolayer as the interface linker. *Chem*

Commun 2022;**58**:9266–9.

133. Li L, Wang Y, Wang X *et al.* Flexible all-perovskite tandem solar cells approaching 25% efficiency with molecule-bridged hole-selective contact. *Nat Energy* 2022;**7**:708–17.

134. Deng X, Qi F, Li F *et al.* Co-assembled Monolayers as Hole-Selective Contact for High-Performance Inverted Perovskite Solar Cells with Optimized Recombination Loss and Long-Term Stability. *Angew Chem Int Ed* 2022;**61**:e202203088.

135. Liao Q, Wang Y, Zhang Z *et al.* Self-assembled donor-acceptor hole contacts for inverted perovskite solar cells with an efficiency approaching 22%: The impact of anchoring groups. *J Energy Chem* 2022;**68**:87–95.

136. Aktas E, Pudi R, Phung N *et al.* Role of Terminal Group Position in Triphenylamine-Based Self-Assembled Hole-Selective Molecules in Perovskite Solar Cells. *ACS Appl Mater Interfaces* 2022;**14**:17461–9.

137. Zhang J, Yang J, Dai R *et al.* Elimination of Interfacial Lattice Mismatch and Detrimental Reaction by Self-Assembled Layer Dual-Passivation for Efficient and Stable Inverted Perovskite Solar Cells. *Adv Energy Mater* 2022;**12**:2103674.

138. Kapil G, Bessho T, Sanehira Y *et al.* Tin–Lead Perovskite Solar Cells Fabricated on Hole Selective Monolayers. *ACS Energy Lett* 2022;**7**:966–74.

139. Zheng L, Xuan Y, Wang J *et al.* Inverted perovskite/silicon V-shaped tandem solar cells with 27.6% efficiency via self-assembled monolayer-modified nickel oxide layer. *J Mater Chem A* 2022;**10**:7251–62.

140. Liu H, Yan K, Rao J *et al.* Self-Assembled Donor–Acceptor Dyad Molecules Stabilize the Heterojunction of Inverted Perovskite Solar Cells and Modules. *ACS Appl Mater Interfaces* 2022;**14**:6794–800.

141. Zuo X, Kim B, Liu B *et al.* Passivating buried interface via self-assembled novel sulfonium salt toward stable and efficient perovskite solar cells. *Chem Eng J* 2022;**431**:133209.

142. Ullah A, Park KH, Nguyen HD *et al.* Novel Phenothiazine-Based Self-Assembled Monolayer as a Hole Selective Contact for Highly Efficient and Stable p-i-n

Perovskite Solar Cells. *Adv Energy Mater* 2022;**12**:2103175.

143. Zhang H, Li K, Sun M *et al.* Design of Superhydrophobic Surfaces for Stable Perovskite Solar Cells with Reducing Lead Leakage. *Adv Energy Mater* 2021;**11**:2102281.

144. Li S, Lu H, Kan Z *et al.* Engineering of P3CT-Na through diprophylline treatment to realize efficient and stable inverted perovskite solar cells. *Chem Eng J* 2021;**419**:129581.

145. Kim SY, Kang H, Chang K *et al.* Case Studies on Structure–Property Relations in Perovskite Light-Emitting Diodes via Interfacial Engineering with Self-Assembled Monolayers. *ACS Appl Mater Interfaces* 2021;**13**:31236–47.

146. Aktas E, Phung N, Köbler H *et al.* Understanding the perovskite/self-assembled selective contact interface for ultra-stable and highly efficient p–i–n perovskite solar cells. *Energy Environ Sci* 2021;**14**:3976–85.

147. Wang G, Wang C, Gao Y *et al.* Passivation agent with dipole moment for surface modification towards efficient and stable perovskite solar cells. *J Energy Chem* 2022;**64**:55–61.

148. Mann DS, Patil P, Kwon S-N *et al.* Enhanced performance of p-i-n perovskite solar cell via defect passivation of nickel oxide/perovskite interface with self-assembled monolayer. *Appl Surf Sci* 2021;**560**:149973.

149. Dai Z, Yadavalli SK, Chen M *et al.* Interfacial toughening with self-assembled monolayers enhances perovskite solar cell reliability. *Science* 2021;**372**:618–22.

150. Shi Y, Zhang H, Tong X *et al.* Interfacial Engineering via Self-Assembled Thiol Silane for High Efficiency and Stability Perovskite Solar Cells. *Sol RRL* 2021;**5**:2100128.

151. Chang C-Y, Huang H-H, Tsai H *et al.* Facile Fabrication of Self-Assembly Functionalized Polythiophene Hole Transporting Layer for High Performance Perovskite Solar Cells. *Adv Sci* 2021;**8**:2002718.

152. Al-Ashouri A, Köhnen E, Li B *et al.* Monolithic perovskite/silicon tandem solar cell with >29% efficiency by enhanced hole extraction. *Science*

2020;**370**:1300–9.

153. Kim G-W, Choi Y, Choi H *et al.* Novel cathode interfacial layer using creatine for enhancing the photovoltaic properties of perovskite solar cells. *J Mater Chem A* 2020;**8**:21721–8.

154. Shu H, Xia J, Yang H *et al.* Self-Assembled Hydrophobic Molecule-Based Surface Modification: A Strategy to Improve Efficiency and Stability of Perovskite Solar Cells. *ACS Sustain Chem Eng* 2020;**8**:10859–69.

155. Más-Montoya M, Gómez P, Curiel D *et al.* A Self-Assembled Small-Molecule-Based Hole-Transporting Material for Inverted Perovskite Solar Cells. *Chem – Eur J* 2020;**26**:10276–82.

156. Anizelli H, David TW, Tyagi P *et al.* Enhancing the stability of perovskite solar cells through functionalisation of metal oxide transport layers with self-assembled monolayers. *Sol Energy* 2020;**203**:157–63.

157. Tumen-Ulzii G, Matsushima T, Klotz D *et al.* Hysteresis-less and stable perovskite solar cells with a self-assembled monolayer. *Commun Mater* 2020;**1**:1–7.

158. Yan J, Lin Z, Cai Q *et al.* Choline Chloride-Modified SnO2 Achieving High Output Voltage in MAPbI3 Perovskite Solar Cells. *ACS Appl Energy Mater* 2020;**3**:3504–11.

159. Wolff CM, Canil L, Rehermann C *et al.* Perfluorinated Self-Assembled Monolayers Enhance the Stability and Efficiency of Inverted Perovskite Solar Cells. *ACS Nano* 2020;**14**:1445–56.

160. Han J, Kwon H, Kim E *et al.* Interfacial engineering of a ZnO electron transporting layer using self-assembled monolayers for high performance and stable perovskite solar cells. *J Mater Chem A* 2020;**8**:2105–13.

161. Liu C, Zhang D, Li Z *et al.* Incorporating a Polar Molecule to Passivate Defects for Perovskite Solar Cells. *Sol RRL* 2020;**4**:1900489.

162. Zhu T, Su J, Labat F *et al.* Interfacial Engineering through Chloride-Functionalized Self-Assembled Monolayers for High-Performance Perovskite Solar Cells. *ACS Appl Mater Interfaces* 2020;**12**:744–52.

163. Li L, Wu Y, Li E *et al.* Self-assembled naphthalimide derivatives as an efficient and low-cost electron extraction layer for n-i-p perovskite solar cells. *Chem Commun* 2019;**55**:13239–42.

164. Al-Ashouri A, Magomedov A, Roß M *et al.* Conformal monolayer contacts with lossless interfaces for perovskite single junction and monolithic tandem solar cells. *Energy Environ Sci* 2019;**12**:3356–69.

165. Hsieh H-C, Hsiow C-Y, Su Y-A *et al.* Two-dimensional polythiophene homopolymer as promising hole transport material for high-performance perovskite solar cells. *J Power Sources* 2019;**426**:55–60.

166. Maity S, Das B, Maity R *et al.* Improvement of quantum and power conversion efficiency through electron transport layer modification of ZnO/perovskite/PEDOT: PSS based organic heterojunction solar cell. *Sol Energy* 2019;**185**:439–44.

167. Han F, Hao G, Wan Z *et al.* Bifunctional electron transporting layer/perovskite interface linker for highly efficient perovskite solar cells. *Electrochim Acta* 2019;**296**:75–81.

168. Yalcin E, Can M, Rodriguez-Seco C *et al.* Semiconductor self-assembled monolayers as selective contacts for efficient PiN perovskite solar cells. *Energy Environ Sci* 2019;**12**:230–7.

169. Han F, Tu Z, Wan Z *et al.* Effect of functional group position change of pyridinesulfonic acid as interface-modified layer on perovskite solar cell. *Appl Surf Sci* 2018;**462**:517–25.

170. Magomedov A, Al-Ashouri A, Kasparavičius E *et al.* Self-Assembled Hole Transporting Monolayer for Highly Efficient Perovskite Solar Cells. *Adv Energy Mater* 2018;**8**:1801892.

171. Guo X, Zhang B, Lin Z *et al.* Interface engineering of TiO2/perovskite interface via fullerene derivatives for high performance planar perovskite solar cells. *Org Electron* 2018;**62**:459–67.

172. Hou M, Zhang H, Wang Z *et al.* Enhancing Efficiency and Stability of Perovskite Solar Cells via a Self-Assembled Dopamine Interfacial Layer. *ACS Appl Mater*

Interfaces 2018;**10**:30607–13.

173. Akın Kara D, Kara K, Oylumluoglu G *et al.* Enhanced Device Efficiency and Long-Term Stability via Boronic Acid-Based Self-Assembled Monolayer Modification of Indium Tin Oxide in a Planar Perovskite Solar Cell. *ACS Appl Mater Interfaces* 2018;**10**:30000–7.

174. Azmi R, Hadmojo WT, Sinaga S *et al.* High-Efficiency Low-Temperature ZnO Based Perovskite Solar Cells Based on Highly Polar, Nonwetting Self-Assembled Molecular Layers. *Adv Energy Mater* 2018;**8**:1701683.

175. Will J, Hou Y, Scheiner S *et al.* Evidence of Tailoring the Interfacial Chemical Composition in Normal Structure Hybrid Organohalide Perovskites by a Self-Assembled Monolayer. *ACS Appl Mater Interfaces* 2018;**10**:5511–8.
